# Excessive Cell Growth Causes Cytoplasm Dilution And Contributes to Senescence

**DOI:** 10.1016/j.cell.2019.01.018

**Published:** 2019-02-21

**Authors:** Gabriel E. Neurohr, Rachel L. Terry, Jette Lengefeld, Megan Bonney, Gregory P. Brittingham, Fabien Moretto, Teemu P. Miettinen, Laura Pontano Vaites, Luis M. Soares, Joao A. Paulo, J. Wade Harper, Stephen Buratowski, Scott Manalis, Folkert J. van Werven, Liam J. Holt, Angelika Amon

**Affiliations:** 1 David H. Koch Institute for Integrative Cancer Research, Howard Hughes Medical Institute, Massachusetts Institute of Technology, Cambridge, MA 02139, USA; 2 Department of Biology, Massachusetts Institute of Technology, Cambridge, MA 02139, USA; 3 Department of Systems Biology, Harvard Medical School, Boston, Massachusetts 02115, USA; 4 Institute for Systems Genetics, New York University Langone Health, New York, NY 10016, USA; 5 Cell Fate and Gene Regulation Laboratory, The Francis Crick Institute, 1 Midland Road, NW1 1AT London, UK; 6 David H. Koch Institute for Integrative Cancer Research, Massachusetts Institute of Technology, Cambridge, MA 02139, USA; 7 Department of Biological Engineering, Massachusetts Institute of Technology, Cambridge, MA 02139, USA; 8 Department of Mechanical Engineering, Massachusetts Institute of Technology, Cambridge, MA 02139, USA; 9 Department of Cell Biology, Harvard Medical School, Boston, MA 02115, USA; 10 Department of Biological Chemistry and Molecular Pharmacology, Harvard Medical School, Boston, MA 02115, USA; 11 MRC Laboratory for Molecular Cell Biology, University College London, Gower Street, London, WC1E 6BT, UK; 12 Novartis Institute for Biomedical Research, Oncology Department, Cambridge, MA 02139

## Abstract

Cell size varies greatly between cell types, yet within a specific cell type and growth condition, cell size is narrowly distributed. Why maintenance of a cell-type specific cell size is important remains poorly understood. Here we show that growing budding yeast and primary mammalian cells beyond a certain size impairs gene induction, cell-cycle progression, and cell signaling. These defects are due to the inability of large cells to scale nucleic acid and protein biosynthesis in accordance with cell volume increase, which effectively leads to cytoplasm dilution. We further show that loss of scaling beyond a certain critical size is due to DNA becoming limiting. Based on the observation that senescent cells are large and exhibit many of the phenotypes of large cells, we propose that the range of DNA:cytoplasm ratio that supports optimal cell function is limited and that ratios outside these bounds contribute to aging.

## Highlights

• Deviation from normal cell size interferes with cell function and proliferation
• DNA becomes limiting for cell function if cells grow too large
• Uncoupling of protein synthesis and volume causes cytoplasm dilution in big cells
• Excessive cell growth contributes to functional decline in senescence

## Graphical Abstract

**Figure:**
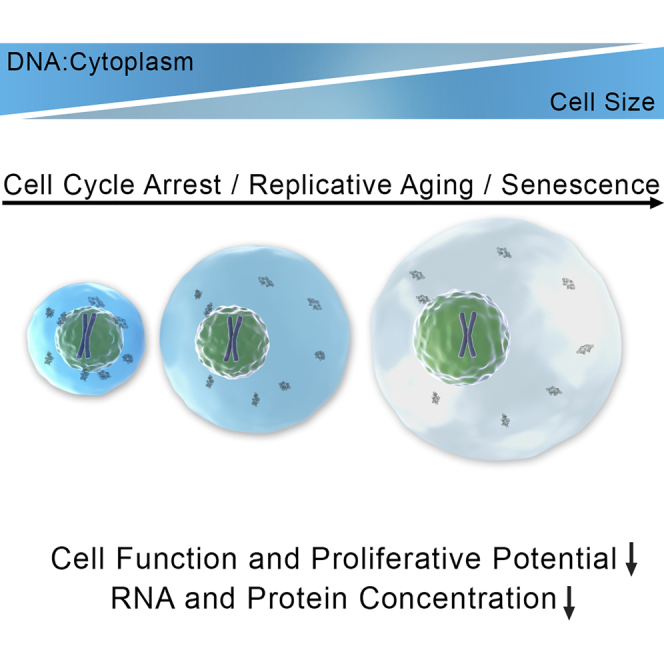

## Teaser

Optimal cell function requires maintenance of a narrow range of DNA:cytoplasm ratios and when cell size exceeds this ratio cytoplasmic dilution contributes to senescence

## Introduction

In multicellular organisms, cell size ranges over several orders of magnitude. This is most extreme in gametes and polyploid cells but is also seen in diploid somatic cells and unicellular organisms. While cell size varies greatly between cell types, size is narrowly constrained for a given cell type and growth condition, suggesting that a specific size is important for cell function. Indeed, changes in cell size are often observed in pathological conditions such as cancer, with tumor cells frequently being smaller and heterogeneous in size (Ginzberg et al., 2015, Lloyd, 2013). Cellular senescence in human cell lines and budding yeast cells is also associated with a dramatic alteration in size. Senescing cells becoming exceedingly large (Hayflick and Moorhead, 1961, Mortimer and Johnston, 1959).

Cell size control has been studied extensively in a number of different model organisms. In budding yeast, cells pass from G1 into S phase, a cell-cycle transition also known as START, at a well-defined cell size that depends on genotype and growth conditions (Turner et al., 2012). Cell growth and division are, however, only loosely entrained. When cell-cycle progression is blocked either by chemical or genetic perturbations cells continue to increase in size (Demidenko and Blagosklonny, 2008, Johnston et al., 1977). During prolonged physiological cell-cycle arrest mechanisms appear to be in place that ensure that they do not grow too large. In budding yeast, for example, mating requires that cells arrest in G1. Cell growth is significantly attenuated during this prolonged arrest by actin polarization-dependent downregulation of the TOR pathway (Goranov et al., 2013). This observation suggests that preventing excessive cell growth is important. Why cell size may need to be tightly regulated is not known.

Several considerations argue that altering cell size is likely to have a significant impact on cell physiology. Changes in cell size affect intracellular distances, surface to volume ratio and DNA:cytoplasm ratio. It appears that cells adapt to changes in cell size, at least to a certain extent. During the early embryonic divisions in *C. elegans*, as cell size decreases rapidly, spindle size shrinks accordingly (Hara and Kimura, 2009). Other cellular structures such as mitotic chromosomes, the nucleus and mitochondria have also been observed to scale with size in various organisms (Levy and Heald, 2012, Neurohr et al., 2011). Similarly, gene expression scales with cell size in human cell lines as well as in yeast (Marguerat et al., 2012, Padovan-Merhar et al., 2015, Zhurinsky et al., 2010). However, not all cellular pathways can adapt to changes in cell size. For example, signaling through the spindle assembly checkpoint, a surveillance mechanism that ensures that cells enter anaphase only after all chromosomes have attached to the mitotic spindle, is less efficient in large cells in *C. elegans* embryos (Galli and Morgan, 2016). In human cell lines, maximal mitochondrial activity is only achieved at an optimal cell size (Miettinen and Björklund, 2016). Finally, large cell size has been shown to impair cell proliferation in budding yeast and human cell lines (Demidenko and Blagosklonny, 2008, Goranov et al., 2013).

Here we identify the molecular basis of the defects observed in cells that have grown too big. We show that in large yeast and human cells, RNA and protein biosynthesis does not scale in accordance with cell volume, effectively leading to dilution of the cytoplasm. This lack of scaling is due to DNA becoming rate-limiting. We further show that senescent cells, which are large, exhibit many of the phenotypes of large cells. We conclude that maintenance of a cell type-specific DNA:cytoplasm ratio is essential for many, perhaps all, cellular processes and that growth beyond this cell type-specific ratio contributes to senescence.

## Results

### A System to Increase Cell Size without Altering DNA Content

**Figure 1:**
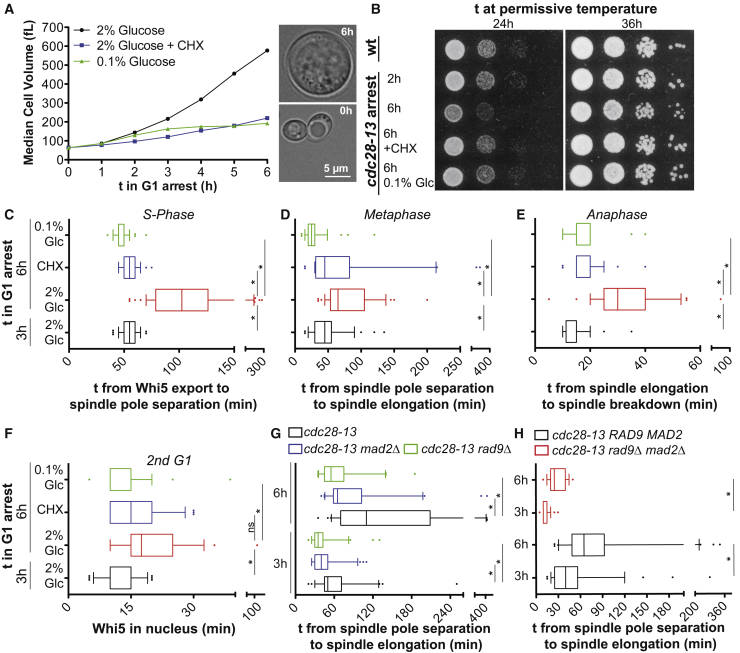
Large Cell Size Impairs Cell Proliferation (A) *Left:* Logarithmically growing *cdc28-13* cells were shifted to 37°C under the indicated growth conditions (CHX = cycloheximide) and volume was determined using a coulter counter. *Right:* Representative images of a cell before and 6 h after shift to 37°C grown in 2% glucose. (B) 10-fold serial dilutions of *cdc28-13* cells arrested at 37°C as indicated were plated and grown at 25°C. (C–H) *cdc28-13* cells expressing Whi5-tdTomato and Spc42-GFP (C–E, G, H) or *CLN2pr*-GFP (F) were arrested at 37°C under the indicated conditions. Cells were shifted to medium containing 2% glucose lacking drugs at 25°C and cell-cycle progression was monitored. Asterisks indicate p < 0.05 (Mann-Whitney U test). See also Figures S1, S2, S3.

We took advantage of the fact that cell growth continues during cell-cycle arrests to alter cell size without changing DNA content. We employed two different temperature sensitive alleles of *CDC28* to reversibly arrest budding yeast cells in G1: *cdc28-13* and *cdc28-4*. Among all the *cdc* mutants, these *CDC28* alleles provided us with the greatest dynamic range to explore the effects of altering cell size on cellular physiology (Goranov et al., 2009). Within 6 h of growth at the restrictive temperature, cells harboring the temperature sensitive *cdc28-13* allele increase their volume almost 10-fold from 65 fL to 600 fL; *cdc28-4* mutants reach sizes of up to 800 fL (Figure 1A and data not shown).

To distinguish between phenotypes caused by a prolonged G1 arrest and phenotypes that are a consequence of increased cell size, we generated two G1 arrested cell populations in all our experiments: (1) Cells that were arrested in G1 and allowed to grow to their maximal size and (2) cells that were arrested in G1 but were prevented from growing large by addition of a low concentration of cycloheximide or by limiting glucose (Figure 1A). Comparing the two populations allowed us to assess the phenotypic consequences of an exceedingly large cell size, as opposed to changes associated with prolonged cell-cycle arrest.

### Increased Cell Size Impairs Cell-Cycle Progression

The G1 arrest caused by the *cdc28-13* allele is reversible: cells re-enter the cell cycle upon return to the permissive temperature (25°C; Marini and Reed, 1992). We found that cells grown large during the G1 arrest resumed proliferation more slowly than small cells upon downshift to 25°C, as judged by colony formation (Figure 1B; Goranov et al., 2013). The number of colonies produced by large cells was similar to that of small cells, indicating that cells did not die during the arrest but proliferated more slowly upon return to the permissive temperature.

**Figure S1:**
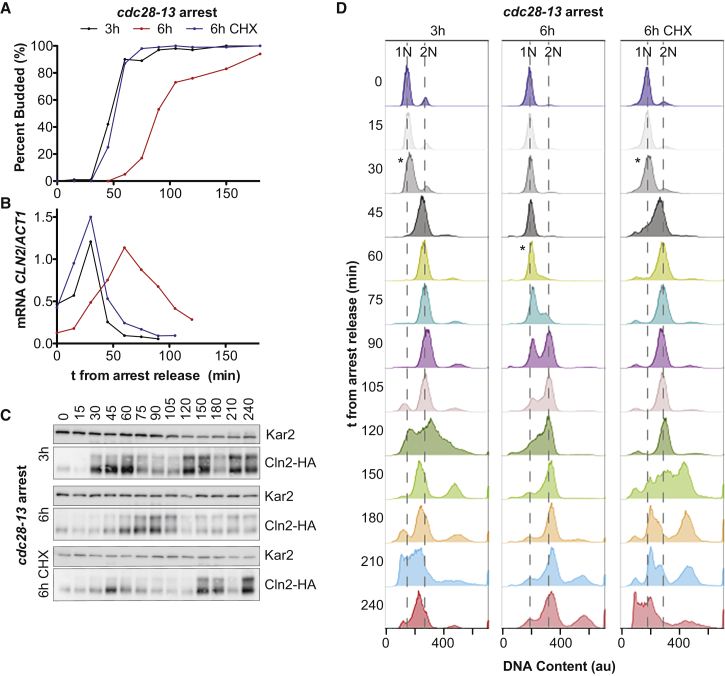
Increased Cell Size Delays Cell-Cycle Progression, Related to Figure 1 (A–D) Logarithmically growing *cdc28-13* mutant cells expressing a Cln2-HA fusion protein were arrested at 37°C as indicated (CHX = cycloheximide). Cultures were shifted to 25°C in the absence of drugs and samples were taken every 15 min. (A) Percentage of budded cells (100 cells per sample). (B) Abundance of *CLN2* mRNA was determined by RT-qPCR and normalized to *ACT1* mRNA. (C) Western blot analysis with antibodies against the HA epitope and Kar2 (loading control). (D) DNA content was analyzed by flow cytometry. Asterisks indicate the time point with maximal *CLN2* mRNA expression.

To investigate cell-cycle defects in large cells we analyzed cell-cycle progression by bud formation, DNA replication, and expression of the G1 cyclin Cln2. Cells arrested for 6 h in G1 progressed into S phase more slowly. This delay was a consequence of increased cell size as cells arrested in G1 in the presence of cycloheximide entered S phase more rapidly upon release from the G1 arrest (Figures S1A–S1C). DNA replication was also delayed in large cells (Figure S1D).

Because release from the G1 arrest was asynchronous in large cells, we turned to live cell imaging to further characterize their cell-cycle defects. We generated cells expressing the fusion proteins Spc42-GFP and Whi5-tdTomato. Spc42 is a component of the spindle pole body (SPB). The appearance of two clearly separated Spc42-GFP foci in cells marks the assembly of a short spindle in late S phase. Fast steady movement of the two SPBs away from each other signals anaphase onset, cessation thereof mitotic spindle breakdown (Winey and O’Toole, 2001). The Whi5-tdTomato fusion resides in the nucleus throughout G1 and leaves the nucleus when cells enter the cell cycle, at START (Costanzo et al., 2004). Live cell analysis of cells harboring both fusions demonstrated that all cell-cycle stages analyzed were delayed in cells that grew to 600 fL during the 6 h G1 arrest (Figures 1C–1F). These delays were due to large cell size. Upon release from the G1 arrest, cells grown in medium containing cycloheximide or low amounts of glucose for 6 h progressed through the cell cycle with kinetics similar to that of cells that were arrested in G1 for only 3 h (Figures 1C–1F).

**Figure S2:**
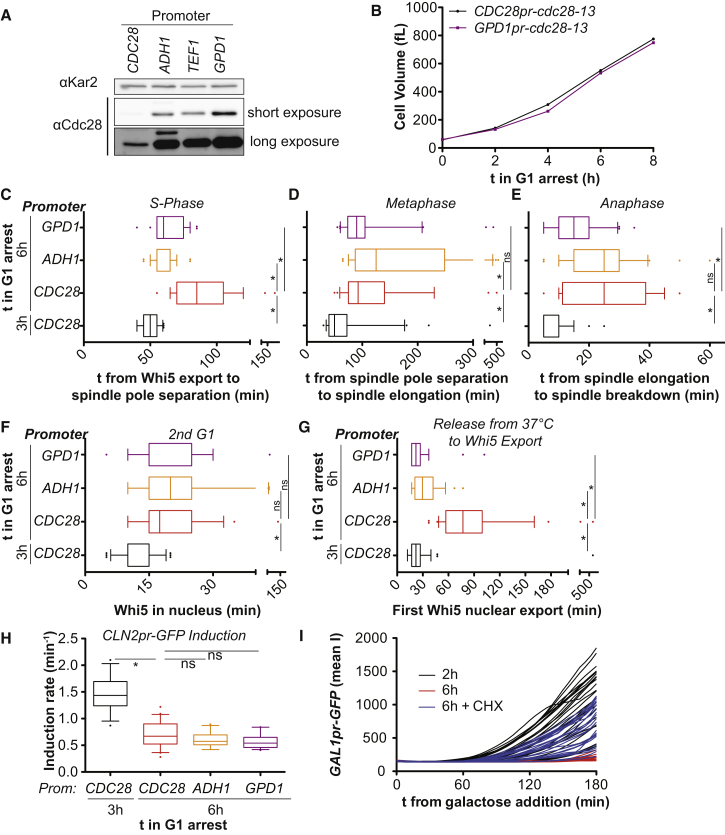
Overexpression of *cdc28-13* Advances Release from G1 Arrest but Does Not Affect Other Large Size Phenotypes, Related to Figure 1 (A) Western blot analysis of Cdc28-13 expressed from the indicated promoters. Kar2 was used as a loading control. (B) Cell volume measured during a G1 arrest in cells expressing Cdc28-13 from the *GPD1* promoter and from the endogenous *CDC28* promoter. (C–H) Cells expressing Cdc28-13 from the indicated promoter were arrested at 37°C for the indicated time, shifted to 25°C and imaged. (C–E) Cells expressing Whi5-tdTomato and Spc42-GFP. (F–H) Cells expressing Whi5-tdTomato and *CLN2pr-*GFP. In (F, H), the data shown for cells expressing Cdc28-13 from its endogenous promoter are the same as those shown in Figures 1F and 2B and are shown here for comparison. (I) *GPD1pr-cdc28-13* cells expressing *GAL1pr-*GFP were arrested as indicated in YEPR (2% raffinose). *GAL1pr-*GFP was induced by addition of 1% galactose and induction was quantified microscopically. Asterisks indicate p < 0.05 (Mann-Whitney U test).

In this analysis we employed a temperature sensitive Cdc28 protein, which upon return to the permissive temperature, needs to be refolded and perhaps resynthesized for cells to resume proliferation (Marini and Reed, 1992). It was possible that some of the cell-cycle defects observed in large cells resulted from Cdc28 activity being limiting. To test this possibility, we expressed *cdc28-13* from the strong, constitutive *ADH1* or *GPD1* promoters to dramatically increase Cdc28-13 levels in cells (Figure S2A). Overexpression of Cdc28-13 did not interfere with cell-cycle arrest and cell growth (Figure S2B), but partially suppressed the cell-cycle entry delay of large cells (Figures S2C and S2G). In contrast, the size associated metaphase and anaphase delay, as well as the G1 delay in the subsequent cell cycle were largely independent of Cdc28-13 protein levels (Figures S2D–S2F). We conclude that replenishing active Cdc28-13 following release from the G1 arrest takes longer in large cells and contributes to the initial cell-cycle entry delay of oversized cells. In contrast, slowed progression through subsequent cell-cycle stages including the next G1 phase are mediated by other aspects of increased cell size.

**Figure S3:**
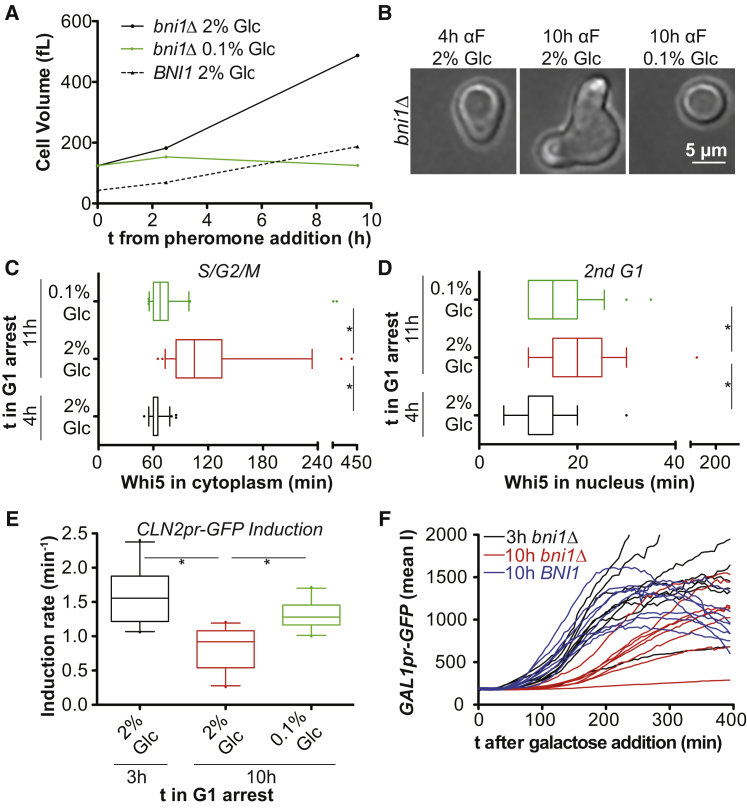
Cell Size Associated Defects Associated with Prolonged Alpha Factor Arrest of Cells Lacking *BNI1*, Related to Figure 1 (A and B) *BNI1* and *bni1Δ* cells (both *bar1Δ*) were arrested in G1 using alpha factor (2 μg/mL) in YEP medium supplemented with either 2% or 0.1% glucose. (A) Cell volume determined on a coulter counter. (B) Representative images of cells treated with alpha factor for the indicated times. (C–E) *bni1Δ* cells expressing Whi5-tdTomato and *CLN2pr*-GFP were arrested in G1 with alpha factor as indicated. Alpha factor was removed and cell-cycle progression was analyzed. *bni1Δ* cells arrested for 4 h with alpha factor had exported Whi5 out of the nucleus before the start of imaging. Cell-cycle phases were therefore measured as cells progressed through the second cell cycle after release from the pheromone arrest. Asterisks indicate p < 0.05 (Mann-Whitney U test). (F) *bni1Δ* and *BNI1* cells expressing *GAL1pr-*GFP were arrested as indicated in YEPR (2% raffinose) and *GAL1pr-GFP* was induced by addition of 1% galactose. Induction was quantified microscopically.

To examine the effects of cell size on cell-cycle progression in a system that did not rely on the use of a conditional *CDC28* allele, we created large cells by arresting *bni1Δ* cells in G1 with α-factor pheromone. During pheromone exposure in wild-type cells, cell growth is restricted to the mating projection due to actin polarization, which reduces the activity of the mTOR pathway (Goranov et al., 2013). Inactivation of the formin Bni1 prevents actin polarization and allows cells to grow large (Figure S3A and S3B; (Goranov et al., 2013)). Upon release from the pheromone-induced G1 arrest, *bni1Δ* cells exhibited cell-cycle delays comparable to those observed in cells grown large due to inactivation of *CDC28* (Figures S3C and S3D). Importantly, restricting growth during the arrest by limiting glucose suppressed these cell-cycle defects (Figures S3C and S3D). Based on results obtained with three different methods to generate large cells, we conclude that increased cell size affects all cell-cycle stages analyzed.

### Checkpoint Activation Delays Cell-Cycle Progression in Oversized Cells

What causes the S phase and mitosis defects in large cells? We hypothesized that DNA replication and/or attachment of chromosomes to the spindle were defective in large cells leading to activation of the DNA damage and spindle assembly checkpoints (SAC), respectively. Indeed, deletion of the DNA damage checkpoint gene *RAD9* and the SAC gene *MAD2* either individually or in combination partially suppressed the metaphase delay of large cells (Figures 1G and 1H). We conclude that cell-cycle checkpoint activation contributes to cell-cycle delays in large cells but checkpoint independent defects also impair cell-cycle progression in large cells.

### Cell-Cycle Regulated Gene Expression Is Inefficient in Large Cells

**Figure 2:**
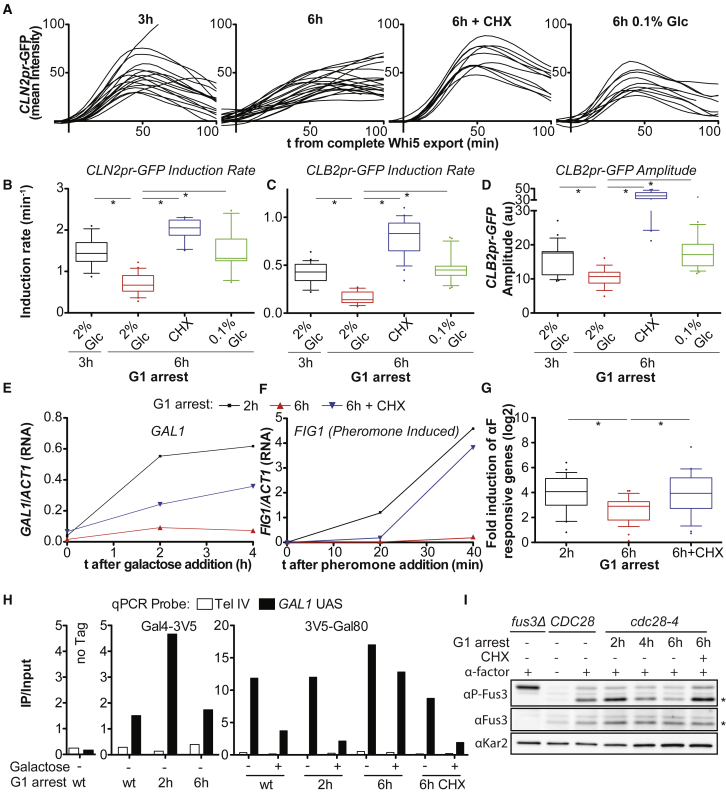
Inducible Transcription Is Impaired in Oversized Cells (A–D) Imaging of cells released from a *cdc28-13* block expressing Whi5-tdTomato and (A, B) *CLN2pr*-GFP or (C, D) *CLB2pr*-GFP into medium containing 2% glucose lacking drugs. Mean GFP intensities were measured on maximal projections and corrected for background and autofluorescence. (A) Traces are aligned when nuclear export of Whi5 was completed. Asterisks indicate p < 0.05 (Mann-Whitney U test). (E–G) *cdc28-4* cells were arrested at 35°C as indicated and transcription was induced by addition of galactose or alpha factor (αF). mRNA concentration was determined by (E, F) RT-qPCR relative to *ACT1* mRNA or by (G) microarray analysis 0 min and 40 min after αF exposure. Genes induced more than 4-fold in wild-type cells were quantified (27 genes). Asterisks indicate p < 0.01 (Wilcoxon matched-pairs signed rank test). (H) Chromatin immuno-precipitation before and 30 min after galactose addition in arrested *cdc28-13* cells, expressing either Gal4-3V5 or 3V5-Gal80. (I) Western blot of phosphorylated Fus3 (P-Fus3) and total Fus3 in *cdc28-4* G1 arrested cells 15 min after pheromone exposure. Kar2 was used as a loading control. Asterisks mark P-Fus3 and Fus3. Note: Fus3 phosphorylation occurs most efficiently during G1. Fus3-P in asynchronously growing cells (lane 3) is therefore lower than in small G1 arrested cells (2 h arrest, lane 4). See also Figure S4 and Table S1.

**Figure S4:**
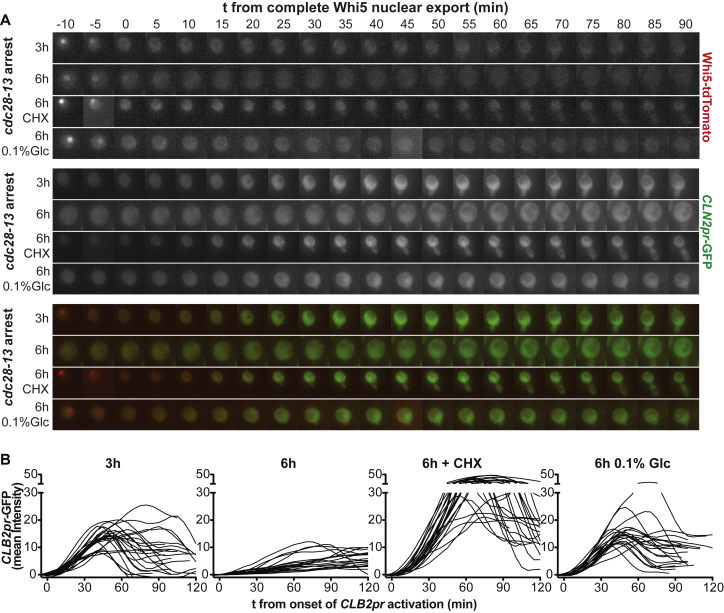
Inefficient Cyclin Induction in Large Cells Causes Checkpoint Independent Cell-Cycle Delays, Related to Figure2 (A) *cdc28-13* cells expressing Whi5-tdTomato and *CLN2pr*-GFP were arrested at 37°C as indicated, shifted to 25°C and imaged. (B) As in A, but cells expressed *CLB2pr*-GFP. Mean GFP intensity was measured on maximal projections and corrected for background and auto-fluorescence. *CLB2pr-*GFP tracks were aligned at the GFP intensity minimum.

The observation that many cell-cycle phases were delayed in large cells suggested that multiple rate-limiting cell-cycle regulators are not produced efficiently in large cells. To test this possibility, we analyzed expression of the G1 cyclin *CLN2* by measuring mean GFP intensities of an unstable GFP protein (GFP-PEST) expressed from the *CLN2* promoter (Mateus and Avery, 2000). We found that *CLN2* was induced more slowly in oversized *cdc28-13* cells, large cells overexpressing Cdc28-13, and pheromone-arrested *bni1Δ* cells, but ultimately GFP levels reached similar amplitudes as in small cells (Figures 2A, 2B, S2H, S3E, S4A). Slow *CLN2* induction was suppressed when cell growth was reduced during the G1 arrest (Figures 2A, 2B, S2H, S3E, S4A).

Analysis of unstable GFP expressed from the mitotic cyclin *CLB2* promoter (*CLB2pr-GFP*, (Mateus and Avery, 2000)) revealed similar results (Figures 2C and S4B). Unlike for *CLN2*, the amplitude of *CLB2* expression was also affected (Figure 2D). We note that when cell growth was inhibited using cycloheximide, both *CLN2pr-GFP* and especially *CLB2pr-GFP* were induced at a faster rate and reached higher amplitudes (Figures 2B–2D, S4A and S4B). Why cycloheximide affects expression of *CLN2* and *CLB2* is presently unknown. We conclude that induction of *CLN2* and *CLB2* is impaired in large cells. We propose that inefficient expression of key cell-cycle regulators is responsible for the checkpoint independent cell-cycle delays observed in large cells.

### Gene Induction in Oversized Cells Is Impaired by Promoter-Specific Mechanisms

Is attenuation of gene induction in large cells restricted to cell-cycle regulated genes or is transcription induction more broadly impacted? To address this question, we analyzed the transcriptional response to changes in nutrient availability and to pheromone exposure in large cells. Induction of the galactose inducible *GAL1* gene and of pheromone induced genes such as *FIG1* was less efficient in cells that grew large during a *cdc28-4* or a *cdc28-13* arrest but not in cells that were kept small during the arrest by cycloheximide treatment (Figures 2E–2G). Similar results were obtained in large cells generated by arresting *bni1Δ* cells with pheromone and in cells where *cdc28-13* was overexpressed (Figure S2, Figure S3I and S3F). We conclude that large cells are defective in transcriptional responses to intra- and extra-cellular cues.

To determine why transcription induction was defective in large cells, we first examined the *GAL1* promoter. Using chromatin immunoprecipitation (ChIP), we determined that the transcriptional repressor Gal80 was not removed from promoters (Figure 2H). As a result, recruitment of the TATA-box binding protein Spt15 and RNA polymerase to the *GAL1* promoter was impaired (data not shown). The defect in pheromone induced gene expression was due to defects in pheromone signaling. Pheromone activates the MAPK Fus3 (Atay and Skotheim, 2017). In oversized cells, Fus3 was phosphorylated less efficiently in response to pheromone (Figure 2I). This finding indicates that the pheromone MAPK signaling cascade is defective in large cells. Together, our results show that pathway specific defects contribute to the observed gene induction defects in oversized cells.

### RNA and Protein Biosynthesis Do Not Scale with Cell Volume in Large Cells

**Figure 3:**
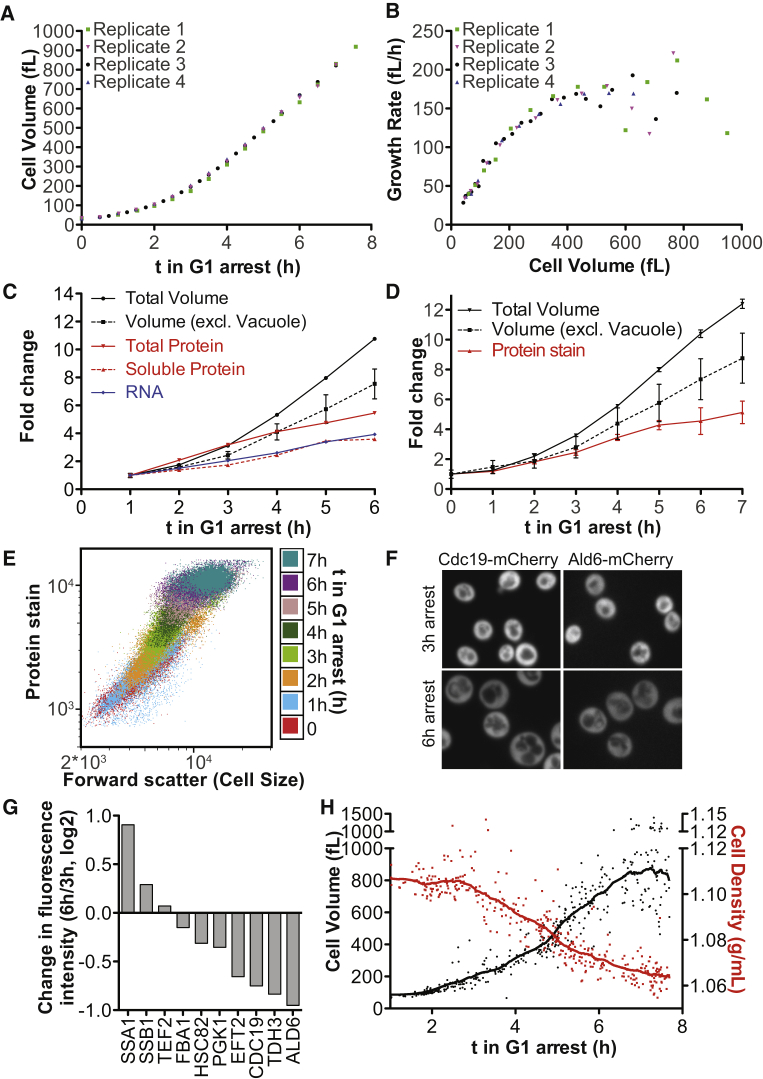
Macromolecule Biosynthesis Does Not Scale with Cell Size (A–C) Newborn *cdc28-13* cells were collected by centrifugal elutriation and arrested at 37°C. (A) Cell volume determined on a coulter counter and (B) growth rate in 4 biological replicates. (C) Volume excluding the vacuole was measured on serial sections of cells expressing Pgk1-mCherry. In an independent experiment, total protein content per cell was determined by Comassie staining of total protein on SDS-PAGE. Soluble protein was determined by Bradford assay in cell lysates prepared without detergent. Total RNA content was measured on a spectrophotometer. (D–G) Logarithmically growing *cdc28-13* cells were arrested at 37°C. (D, E) Cells were fixed and total protein was stained using an amine reactive dye and analyzed by flow cytometry. Cell volume was determined as in (C). (F, G) Cells expressing 10 different mCherry- and GFP- fusion proteins were arrested at 37°C for 3 h and 6 h. Representative images in (F). Mean fluorescence intensity in the cytoplasm in (G). (H) Cell volume and density of individual cells arrested in G1 determined on an SMR. See also Figure S5.

The broad impact of increased size on cell physiology prompted us to investigate whether overall macromolecule biosynthesis was deregulated in large cells. We isolated very small, newly born *cdc28-13* cells (30 fL) by centrifugal elutriation and shifted them to the restrictive temperature (37°C). We then measured cell volume as well as total cellular protein and RNA levels as cells grew bigger during the arrest. As previously reported for dividing budding yeast cells, growth rate was initially proportional to cell volume and thus exponential (Figures 3A and 3B; Cermak et al., 2016). However, once cells grew larger than approximately 200 fL (3 h arrest), growth rates started to plateau resulting in a more linear growth pattern (Figure 3B). Indeed, linear growth was previously observed in large arrested *cdc28-4* cells (Goranov et al., 2009).

**Figure S5:**
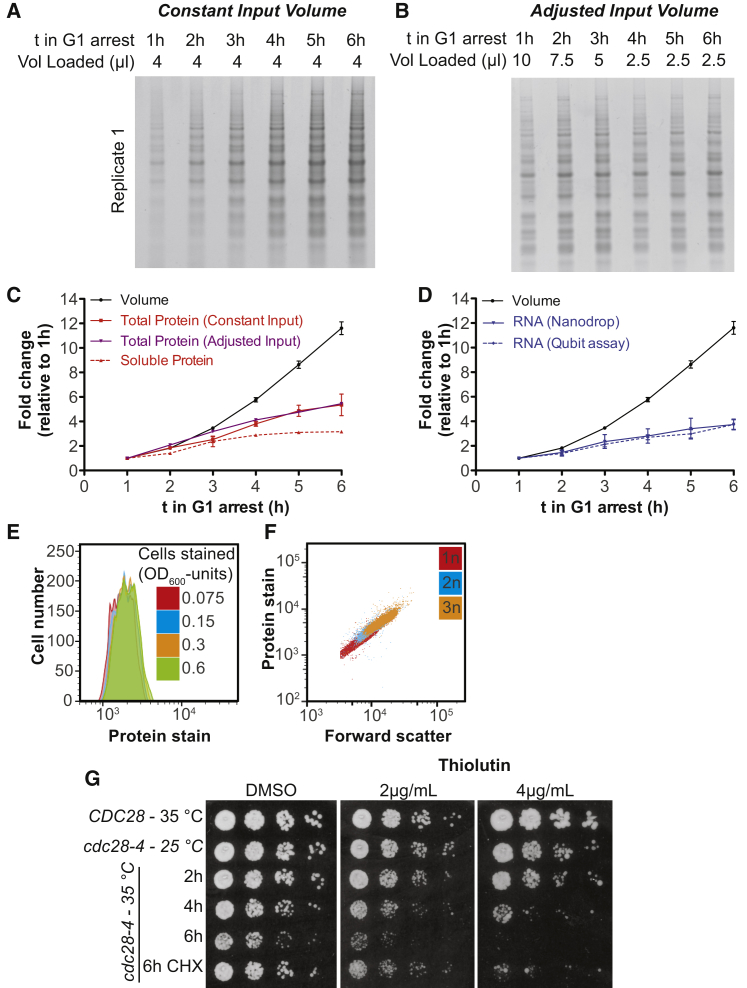
Cellular Protein and RNA Quantification during Prolonged G1 arrest, Related to Figure 3 (A–C) Newborn *cdc28-13* cells were isolated by centrifugal elutriation and arrested in G1 at 37°C. Equal numbers of cells were collected and total protein was isolated by TCA precipitation of cells, followed by mechanical cell lysis in 8M urea and boiling extracts in 3% SDS. (A) Equal volumes of lysate were run on SDS-PAGE followed by Comassie blue staining. Three biological replicates were performed. (B) Different volumes of extract were loaded as indicated to reduce potential effects of non-linearity in the assay. This gel was used to quantify total protein content for Figure 3C. (C) Quantification of total protein from (A, red) and (B, purple). Soluble protein was extracted by breaking cells in Tris/NaCl (without detergent, complete cell lysis was confirmed microscopically) followed by centrifugation (20 min/21000×g) to clear the lysate. 3 biological replicates were analyzed for cells arrested at 37°C for 1 h, 3 h and 5 h. Protein concentration was analyzed using the Bradford protein assay. Cell volume was measured on a coulter counter (the same data for total cell volume, total protein determined with adjusted input and one replicate of soluble protein quantification are shown in Figure 3C). (D) Same experiment as in (A–C), but isolation of total cellular RNA by phenol extraction followed by ethanol precipitation and column purification. Quantifications were performed before (Qubit assay) and after (Nanodrop) column purification of the RNA. (E and F) *cdc28-13* cells were fixed with formaldehyde and subsequently permeabilized in 70% ethanol. Cells were stained with a primary amine reactive dye (Alexa Fluor NHS Ester) to stain total cellular protein and analyzed by flow cytometry. (E) Different numbers of logarithmically growing cells were stained. Cell number is indicated in units of optical density, which correlates with biomass. For logarithmically growing cells, one OD_600_ unit corresponds to roughly 2^∗^10^7^ cells. The same concentration and volume of dye was used for all samples. This analysis shows that the dye does not become limiting. (F) 0.15 OD_600_ units of logarithmically growing haploid (1n), diploid (2n) and triploid (3n) cells were stained. This analysis confirms that this assay can distinguish protein content in differently sized cells. In addition, it shows that in logarithmically growing cells protein content and forward scatter (an estimate of cell size) correlate. (G) *CDC28* and *cdc28-4* mutant cells were arrested at 35°C under the indicated conditions. Ten-fold serial dilutions were plated and grown at 25°C on YEPD (2% glucose) or YEPD supplemented with the pan RNA polymerase inhibitor Thiolutin.

Analysis of total protein, total soluble protein, and total RNA showed that during exponential growth, cell volume increased coordinately with RNA and protein biosynthesis. As cell growth became linear, approximately after 3 h of G1 arrest, RNA and protein accumulated more slowly, in agreement with previous observations in *S. pombe* and theoretical models of cell growth (Lin and Amir, 2018, Zhurinsky et al., 2010). Surprisingly however, cell volume continued to increase at a high rate (Figures 3C, Figures S5A–S5D). This rapid cell volume expansion in the absence of a corresponding increase in RNA and protein biosynthesis was not only driven by a disproportionate increase in vacuolar volume. Measurement of cytoplasmic + nuclear volume by quantifying the volume occupied by the nuclear and cytoplasmic protein Pgk1-mCherry showed that cytoplasmic + nuclear volume increased 7.9-fold between the 1 h and 6 h time points, whereas soluble protein and RNA levels increased by only 3.6-fold and total protein increased 5.5-fold (Figure 3C). Labeling of total cellular protein with an amine reactive dye (Kafri et al., 2013) revealed similar results. Cellular protein content increased 5.1-fold during a 7 h G1 arrest while the volume of the cytoplasm and the nucleus increased by 8.8-fold (Figures 3D, S5E, S5F). Furthermore, the correlation between protein content and cell size (estimated by forward scatter) was lost in very big cells (Figure 3E). To further confirm this result, we chose 10 highly expressed proteins at random, fused them to GFP or mCherry and estimated their protein concentration using confocal microscopy. For 7 of these 10 proteins, we observed a lower concentration in 6 h arrested *cdc28-13* cells compared to 3 h arrested *cdc28-13* cells (Figures 3F and 3G). The two proteins whose abundance increased with size were chaperones of the Hsp70 family (Ssb1 and Ssa1).

Our RNA and protein measurements lead to the remarkable conclusion that, as cells continue to increase in volume during the prolonged G1 arrest, the cyto- and nucleoplasm become diluted. In agreement with this conclusion we found that cell density decreased in large cells as judged by single cell measurements using a suspended micro channel resonator (Figure 3H, Bryan et al., 2010, Son et al., 2015). Cell density declined to 60% of its initial value (relative to the baseline density of water) during a 7 h G1 arrest. This decrease in density dramatically exceeds previously reported fluctuations in cell density that occur during the cell cycle (Baldwin and Kubitschek, 1984, Bryan et al., 2010, Hartwell, 1970). Yeast dry mass is composed of roughly 50% protein, 30% carbohydrates (mostly cell wall), 10% RNA, and 7% lipids and inorganic molecules (Fonseca et al., 2007). When vacuolar volume is included, total protein concentration drops by 53% from its initial value during a 6 h G1 arrest, and RNA concentration by 68%. This predicts a decrease in total cell density by 33%. We measured a 36% drop in cell density. The decrease in RNA and protein concentration can therefore largely explain the decrease in density observed in large cells; substantial loss of carbohydrates and lipids does not appear to occur.

### General Transcription and Translation Factors Do Not Scale with Cell Size

**Figure 4:**
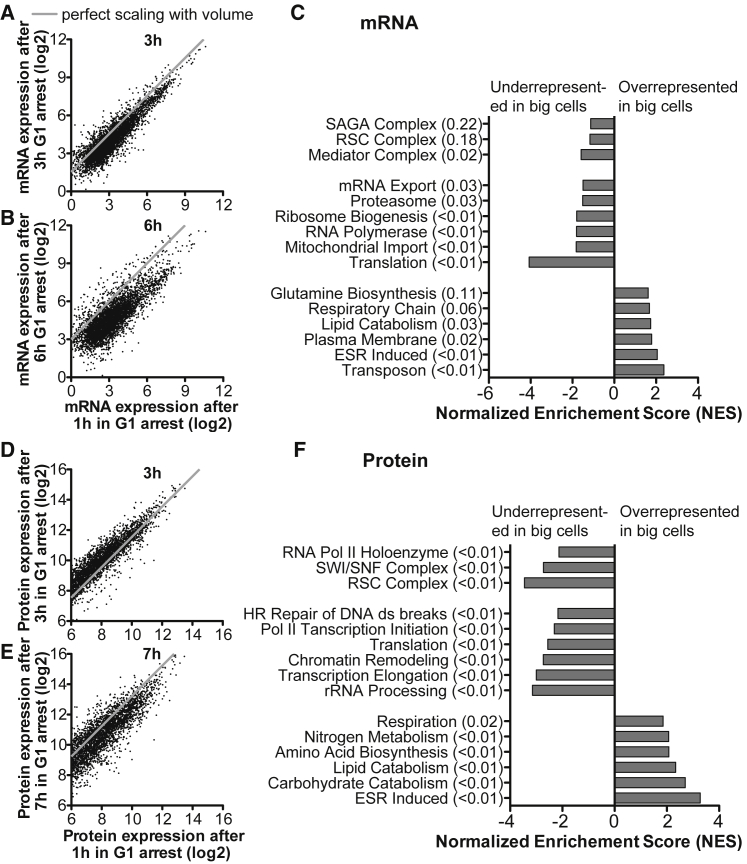
RNASeq and Mass Spec Analysis of Oversized Cells Small *cdc28-13* cells were isolated by centrifugal elutriation and arrested in G1 at 37°C. (A–C) RNA Seq of a constant number of arrested *S. cerevisiae* cells of different sizes mixed with a constant number of exponentially growing *C. albicans* cells before RNA purification. *S. cerevisiae* reads were normalized to total *C. albicans* reads (Units are fragments per kb per million C. albicans reads). RNA levels of cells arrested for 3 h (A) and 6 h (B) at 37°C were compared to RNAs of cells arrested for 1h. (C) Gene set enrichment analysis (GSEA) was performed comparing RNA expression levels from cells arrested for 2 h, 2.5 h and 3 h to expression levels from cells arrested for 4.5 h, 5 h and 6 h at 37°C. False discovery rates are indicated in brackets. (D and E) Proteome of equal numbers of *cdc28-13* cells arrested for 1 h, 3 h, 5 h and 7 h at 37°C was analyzed. 1 h, 3 h and 5 h arrest points were analyzed in triplicate, the 7 h arrest point in duplicate. 3 h (D) and 7 h (E) time point were compared to the 1 h arrest point. (F) GSEA analysis comparing the 3 h and 5 h time points. The gray line in A-B, D-E indicates where individual data points would fall if gene expression level increased proportional to cell volume (excluding vacuole) increase. See also Figure S6 and Tables S2, S3.

Are all RNAs and proteins affected equally by large cells’ inability to scale RNA and protein production with cell volume? To address this question, we performed transcriptome and proteome analyses on differently sized G1- arrested *cdc28-13* cells. We found that during the first 3 h of the G1 arrest, levels of individual mRNAs increased proportionally with cell volume (Figure 4A). Gene expression therefore increases coordinately as previously reported in *S. pombe* (Zhurinsky et al., 2010). By 6 h of G1 arrest however, scaling of most (> 90%) transcripts had ceased (Figure 4B). Gene set enrichment analysis (GSEA, Subramanian et al., 2005) revealed that expression of components of the general transcription and translation machinery did not scale with cell volume. RNAs encoding components of all three RNA polymerases and their cofactors, chromatin remodeling factors important for transcription, and RNAs encoding factors important for ribosome biogenesis and translation were all underrepresented in large cells (Figure 4C). These data suggest that the general transcription and translation machineries become limiting in large cells. Two additional observations support this conclusion. First, large cells are sensitive to the pan-RNA polymerase inhibitor Thiolutin (Figure S5G). Second, many of the genes whose transcripts are selectively lost in large cells are haploinsufficient (Deutschbauer et al., 2005), an indication of these genes being limiting for cell growth and proliferation.

Quantitative proteomic analysis using the tandem mass tag (TMT) multiplexing approach (McAlister et al., 2012) comparing *cdc28-13* cells arrested in G1 for 1 h, 3 h, 5 h and 7 h confirmed that total protein concentration decreased in large cells. Quantification of over 3,800 proteins across the four conditions in triplicate (except the 7 h time point, which was performed in duplicate) showed that while protein content scaled between the 1 h and 3 h arrest points, this was not the case when comparing the 1 h and the 7 h time points (Figures 4D and 4E). During the 7 h arrest, total cytoplasmic and nuclear volume increased 9.4-fold (gray line Figure 4E) but total cellular protein by only 6.2-fold. The proteomic analysis further revealed that loss of RNAs in large cells correlated with the loss of their corresponding proteins. General transcription and translation factors were underrepresented in large cells (Figure 4F). Our analyses show that the lack of scaling between cell volume increase and RNA/protein biosynthesis that occurs once cells exceed a size of 200 fL is caused by limiting transcriptional and translational capacity.

### Oversized Cells Induce a Stress Response

**Figure S6:**
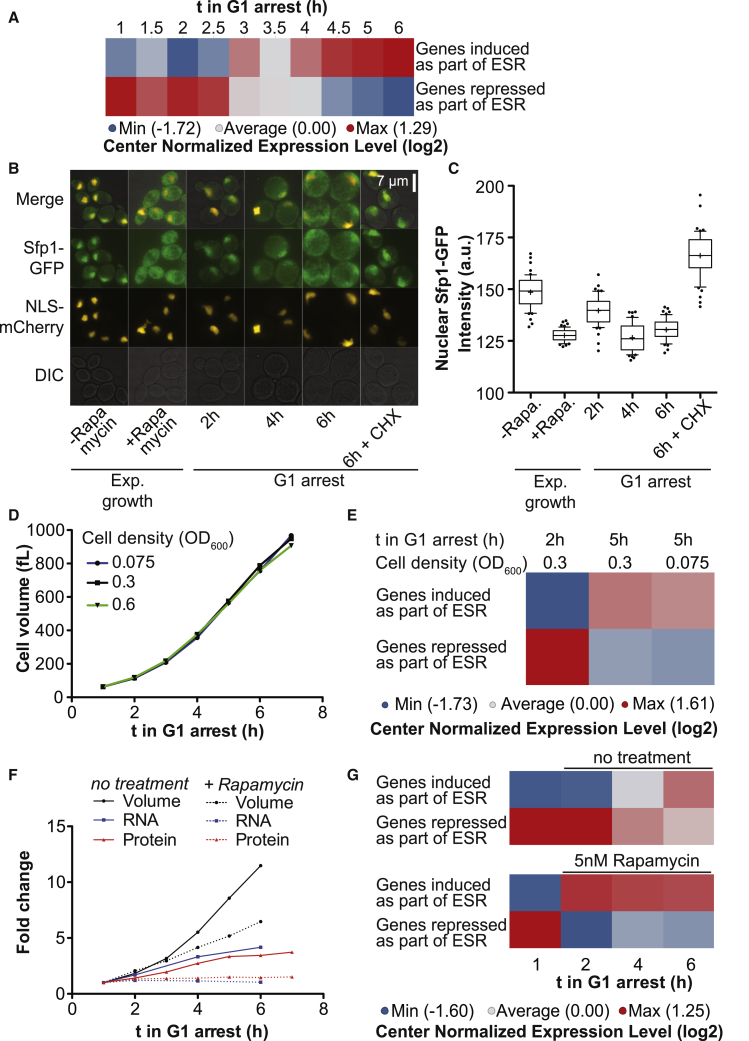
Oversized Cells Induce the Environmental Stress Response (ESR) that Attenuates Macromolecule Synthesis, Related to Figure 4 (A) Data from RNASeq experiment shown in Figure 4. Newborn *cdc28-13* cells were isolated by centrifugal elutriation and arrested at 37°C and processed for RNA Seq. Analysis of relative expression levels of ESR genes: A single sample GSEA projection for 279 stress induced and 584 stress repressed genes was generated for each sample and row centered (see Methods). (B and C) Nuclear localization of Sfp1-GFP was analyzed in *cdc28-4* cells expressing Sfp1-GFP and NLS-mCherry at permissive temperature (asynchronous), after treatment with rapamycin (1 μM for 30 min) and after the indicated times at 35°C. Quantification of mean nuclear Sfp1-GFP intensity is shown in (C). (D and E) The switch from exponential to linear growth and activation of the ESR are not a consequence of low nutrient concentrations in the growth medium after prolonged G1 arrest: Newborn *cdc28-13* cells were isolated by centrifugal elutriation, arrested in G1 at 37°C and diluted to the indicated cell densities 30 min after cell isolation. (D) Cell volume was analyzed on a coulter counter and RNA samples were collected for RNA Seq analysis. (E) ESR strength was determined as described in (A). As comparison, elutriated cells used for other experiments were diluted to an optical density of 0.3 at the same time point of the arrest. (F and G): Newborn *cdc28-13* cells were isolated by centrifugal elutriation and arrested in G1 at 37°C. 1 h after cell isolation, 5 nM Rapamycin was added. Samples were taken for RNA Seq analysis: an equal number of arrested cells was mixed with a constant number of logarithmically growing *C. albicans* cells prior to RNA purification. (F) Total *S. cerevisiae* RNA normalized to total *C. albicans* RNA are shown and cell volume was determined on a coulter counter. Total protein was determined from a different experiment and is shown on the same graph for comparison: *cdc28-13* cells from a logarithmically growing culture were arrested in G1 at 37°C. Cells were fixed and total cellular protein was stained and quantified using flow cytometry. Data points are normalized to the 1 h arrest time point. (G) ESR strength was determined as described in A. All samples (±Rapamycin) were used for center normalization. The data shown for the 1 h time point is the same in the upper and lower panel (prior to Rapamycin addition).

Retro-transposable elements were the most upregulated genes in oversized cells (Figure 4C). Induction of retro-transposition frequently occurs in response to cellular stress (Lesage and Todeschini, 2005). Indeed, cells arrested for longer than 3 h induced a stress response program known as the environmental stress response (ESR; Figure S6A). The ESR is induced in response to a variety of stress conditions and involves the repression of genes required for ribosome biogenesis and, to a lesser extent, general transcription factors (Gasch et al., 2000). We observe this repression also in large cells (Figure 4C). Furthermore, Sfp1, the transcriptional activator that controls ribosome biogenesis (Jorgensen et al., 2004, Marion et al., 2004), is found in the cytoplasm in large cells, unable to promote expression of ribosome biogenesis genes (Figures S6B and S6C). This stress response was caused by increased cell size and not by nutrient limitation in the growth medium, as cultures grown at different cell densities showed no difference in growth rate or ESR activation (Figures S6D and S6E). To test whether ESR activation contributes to cytoplasm dilution, we treated cells with a low concentration (5nM) of the TOR inhibitor Rapamycin. This treatment induced an ESR in small cells and led to cytoplasm dilution in small cells (Figures S6F and S6G), which is in agreement with previous reports (Delarue et al., 2018). We conclude that activation of the ESR contributes to the decoupling of cell volume expansion from RNA and protein biosynthesis in large cells.

### DNA Content Is Rate Limiting in Large Cells

What is ultimately responsible for activation of the ESR and other phenotypes in large cells? Because cells continue to grow in size during a cell-cycle arrest without an accompanying increase in DNA content, DNA could become limiting in large cells. Indeed, DNA becomes limiting for RNA and protein synthesis during prolonged cell-cycle arrests in *S. pombe* (Zhurinsky et al., 2010).

**Figure S7:**
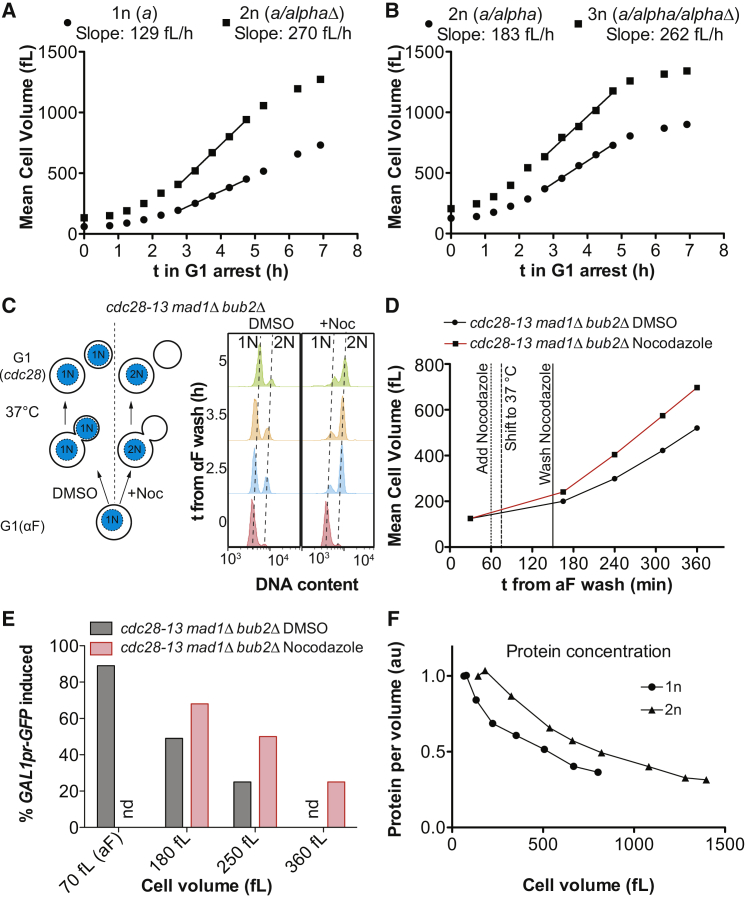
DNA Content Limits Growth Rate and Cell Function in Large Cells, Related to Figure 5 (A and B) Logarithmically growing haploid (1n), diploid (2n) and triploid (3n) cells homozygous for *cdc28-13* were arrested in G1 at 37°C and cell volume was determined on a coulter counter. The genotype at the *MAT* locus is indicated. (C and D) *cdc28-13 mad1Δ bub2Δ* cells were grown in YEPD (2% glucose) and arrested in G1 using alpha factor pheromone for 2 h at 25°C. Subsequently, alpha factor was washed out and cells were released at 25°C into fresh medium. 60 min after alpha factor washout, nocodazole or DMSO were added and 15 min later, cultures were shifted to 37°C. Nocodazole and DMSO were removed 2.5 h after the alpha factor washout. (C) *Left:* Schematic of the experiment. *Right:* DNA content was determined by flow cytometry and (D) cell volume was measured on a coulter counter. (E) *cdc28-13 mad1Δ bub2Δ* mutant cells expressing *GAL1pr-GFP* were treated essentially as described in (C and D) but the arrest was performed in YEPR (2% raffinose) and nocodazole washout was performed at 3.2 h after release from the alpha factor block. *GAL1pr-GFP* expression was induced 4 h, 5 h and 6 h after alpha factor washout at 25°C by addition of 1% galactose. Samples were taken 3 h after galactose addition and GFP expression was analyzed by flow cytometry. Percent of cells that express GFP in equally sized cell populations is shown. Mean cell volume and arrest times were as follows. DMSO treated samples: (180 fL) – 192 fL, 5 h arrest, (250 fL) – 286 fL, 6 h arrest; Nocodazole treated samples: (180 fL) – 183 fL, 4 h arrest, (250 fL) – 256 fL, 5 h arrest, (360 fL) – 363 fL, 6 h arrest. (F) Haploid (1n) and diploid (2n) logarithmically growing *cdc28-13* cells were arrested at 37°C. Cells were fixed and total protein was stained and analyzed on a flow cytometer. Cell volume was determined on a coulter counter. Protein/volume ratio was normalized to logarithmically growing cells.

We analyzed the importance of DNA:cytoplasm ratio by comparing haploid and diploid yeast strains. Diploid *cdc28-13* cells reach a maximal linear growth rate that is 2.1-fold higher than the growth rate of haploid cells; triploid cells grow 1.4-fold faster than diploids (Figures S7A and S7B). To exclude the possibility that the observed differences in growth rate are a consequence of differences in initial cell size and growth rate, we examined cell growth directly after cells had undergone a genome duplication. To induce genome duplication, we treated *cdc28-13* cells that were SAC deficient (they lacked *MAD1* and *BUB2*) with nocodazole, which causes microtubule depolymerization. *mad1Δ, bub2Δ* cells will not arrest in metaphase, but instead will exit from mitosis without dividing their nucleus creating diploid cells (Figure S7C). We synchronized *cdc28-13* cells in G1 using alpha factor, released them into the cell cycle in the presence of nocodazole or DMSO, and arrested cells in the next G1 by shifting them to the restrictive temperature. This growth regiment generated haploid and diploid cells of almost identical cell size from the same initial cell population (Figure S7C). However, cells with a diploid genome content grew faster than haploid cells (Figure S7D), demonstrating that DNA content and not initial cell size determines maximal growth rate.

**Figure 5:**
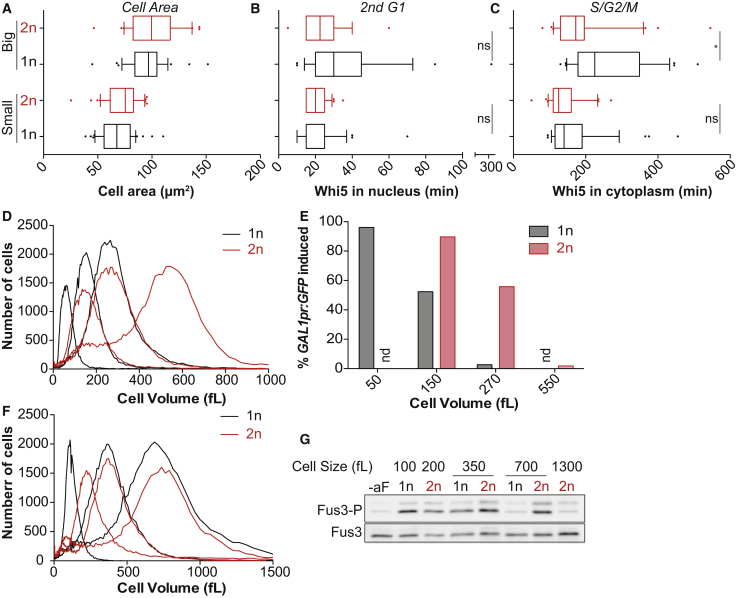
Low DNA:cytoplasm ratio causes large cell phenotypes (A–C) Haploid (1n) and diploid (2n) *cdc28-13* cells expressing Whi5-tdTomato were arrested for different times in G1 to reach an equal cell size (arrest times: 1n: 3 h 30 min, 6 h 15 min; 2n: 2 h 15, 3 h 30 min). Cells were shifted to 25°C and imaged. (D and E) Haploid and diploid *cdc28-13* cells expressing *GAL1pr-*GFP were arrested in raffinose for different amounts of time for cells to reach the same size (arrest times: 1n: 1 h, 4 h, 6 h; 2n: 1 h 30, 3 h, 5 h). *GAL1pr*-GFP was induced by addition of 1% galactose and GFP expression analyzed by FACS 3 h after galactose addition. (F and G) haploid (*MATa*) and diploid (*MATa*/*alphaΔ*) *cdc28-13* cells were arrested for different amounts of time for cells to reach the same size (arrest times: 1n: 1 h 20 min, 3 h 45 min, 6 h 15 min; 2n: 1 h 20 min, 2 h 20 min, 3 h 45 min, 6 h 15 min) and exposed to alpha factor for 5 min to analyze Fus3 phosphorylation. See also Figure S7.

To test whether a decrease in DNA:cytoplasm ratio was responsible for the cellular defects observed in large cells, we analyzed cell-cycle progression, *GAL1* induction and pheromone response in haploid and diploid cells of the same size. We arrested haploid and diploid cells for different times in G1 to obtain equally sized cell populations (Figure 5A, 5D, and 5F). Comparison of haploid and diploid cells of the same size revealed that large diploid cells progressed faster through the cell cycle. *GAL1* promoter induction and pheromone signaling was also more efficient in diploids than haploids of the same large size (Figures 5B, 5C, 5E, and 5G). In fact, large cell phenotypes manifested in diploid cells at twice the size as in haploid cells (Figures 5D–5G). Similarly, cells that had undergone a genome duplication were able to induce *GAL1* at a larger size than cells that had not (Figure S7E). These results demonstrate that the DNA:cytoplasm ratio defines the cell-size range within which RNA and protein biosynthesis occur to the degree necessary to efficiently support dynamic gene expression, cell proliferation and cell signaling. Because cytoplasm dilution occurs at a larger size in diploid cells (Figure S7F) we propose that dilution of the cytoplasm is the underlying cause of the defects caused by large cell size.

### Old Yeast Cells Grow Large and Share Phenotypes with Oversized Young Cells

**Figure 6:**
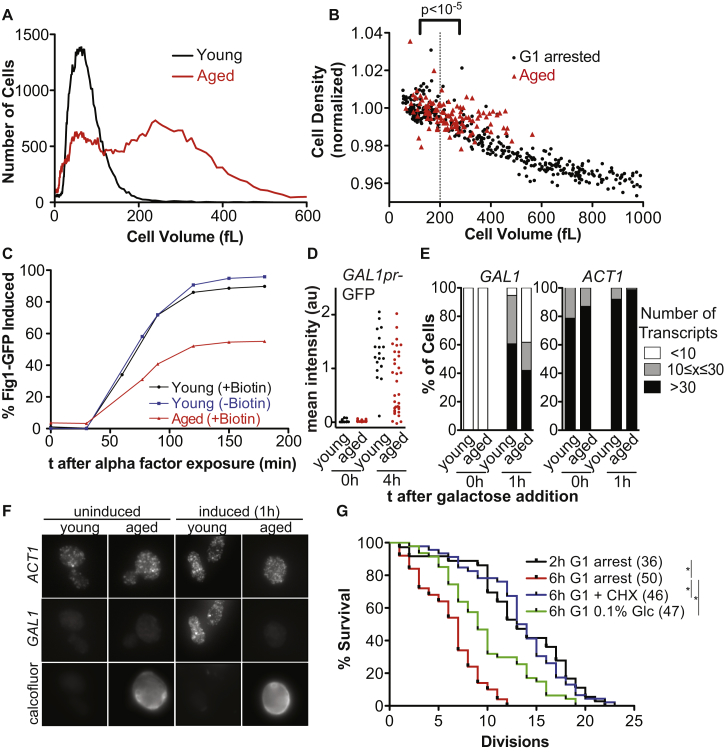
Effects of cell size on replicative aging (A–C) Aged wild-type yeast cells were isolated (50% purity). Average age of old cells was 16.3 ± 2.8 cell divisions, young cells were less than 2 divisions old. (A) Cell volume measured on a coulter counter and (B) cell volume and density measured on an SMR. Data pooled from 3 measurements were normalized to cell density of cells < 200 fL. For comparison, the data of the aged cells are compared to the density of large young cells shown in Figure 3H. (C) Young unlabeled (age: < 2), Young (age: 5.4 ± 1.7) and aged (age: 17.2 ± 1.8) cells labeled with Biotin expressing *FIG1*-GFP (*hmlΔ*) were exposed to 20 μg/mL alpha factor to analyze Fig1-GFP expression (FACS). (D) *GAL1pr*-GFP induction in young (age: 1.6 ± 1.5) and aged (age: 10.5 ± 2.2) cells (microscope). (E, F) Single molecule RNA FISH in cells before (n > 50) or after 1 h (n > 150) of galactose addition (age: young = 2.0 ± 1.4, old = 14.0 ± 2.6). (E) Quantification of *ACT1* mRNA (control) and *GAL1* mRNA. (F) Representative images. Calcofluor staining identifies old cells. (G) Pedigree analysis of *GPD1pr-cdc28-13* expressing cells released from the indicated G1 arrest. Asterisks indicate statistical significant (p < 0.01) median survival (Mann-Whitney U test). Number of cells included in the analysis is shown in brackets.

Are there situations in a budding yeast cell’s life cycle where a cell reaches a size at which RNA and protein biosynthesis become limiting? Because cell size continuously increases during replicative aging (Mortimer and Johnston, 1959), we analyzed cell size in old cells. We found that the majority of old yeast cells (defined as cells that had undergone an average of 16 divisions) was larger than 200 fL, the size beyond which RNA and protein biosynthesis no longer scale with cell volume (Figure 6A). This age-induced cell size increase has been proposed to limit lifespan because cells that are born large due to stochastic events or specific mutations have a reduced lifespan (Yang et al., 2011, Zadrag-Tecza et al., 2009).

Our data show that young cells grown large display a number of phenotypes characteristic of aged cells, including slow cell division, increased DNA damage (data not shown), decreased sensitivity to pheromone and global changes in transcription (Hu et al., 2014, Mortimer and Johnston, 1959, Neurohr et al., 2018, Smeal et al., 1996). This intriguing correlation prompted us to investigate whether other phenotypes uncovered in large cells, and not yet investigated during replicative aging, are also observed in old yeast cells. We found this to be the case. Like in large young cells, density was decreased in old yeast cells (Figure 6B) and old cells were also unable to mount a transcriptional response to pheromone (Figure 6C). We note that this was not due to de-repression of the silenced mating type locus *HML* because impaired induction of pheromone responsive genes was observed in old cells in which the *HML* locus was deleted. Similarly, and like oversized young yeast cells, aged yeast cells were defective in inducing transcription from the *GAL1* promoter (Figures 6D–6F). We conclude that old yeast cells grow to a size at which cytoplasm dilution starts to occur. Consistent with this observation old cells display the same functional defects as oversized young cells.

To move beyond this correlative analysis, we next asked whether increasing cell size was sufficient to limit lifespan. To avoid Cdc28-13 becoming limiting, we used a strain that expressed the protein from the *GPD1* promoter. *GPD1-cdc28-13* cells released from a 2 h G1 block had an average lifespan of 13 generations. Extending the arrest to 6 h reduced the lifespan to 7 generations (Figure 6G). This finding is consistent with previous observations showing that prolonged G1 arrest decreases lifespan (Yang et al., 2011). Importantly, preventing cell volume increase during the G1 arrest, with either cycloheximide or low levels of glucose, restored average lifespan to 13 and 9 generations, respectively. This result demonstrates that an excessive increase in cell size is sufficient to reduce lifespan. We propose that large cell size contributes to multiple phenotypes observed in aged cells. However, we note that cells arrested in G1 for 6 h reach a median size of 600 fL or more, a size well beyond that of old cells. The fact that these 600 fL cells are able to undergo an average of 7 divisions indicates that large cell size can contribute to aging but other factors also determine lifespan in budding yeast.

### Excessive Cell Growth Contributes to Terminal Cell-Cycle Arrest and Reduces Macromolecular Crowding during Senescence in Human Fibroblasts

**Figure 7:**
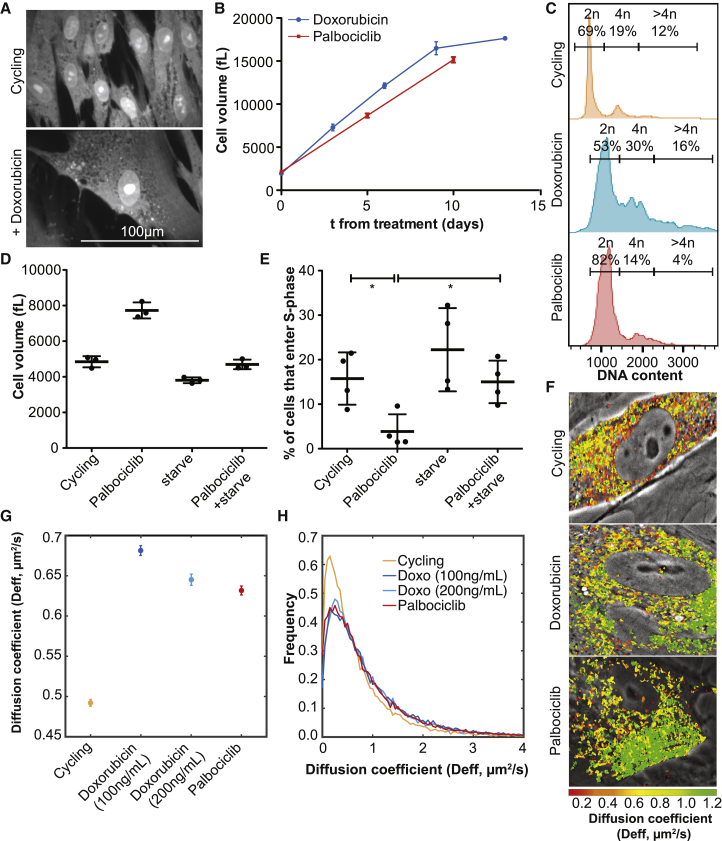
Increased cell size interferes with proliferation in human fibroblasts (A–C) IMR90 cells were treated with Doxorubicin or Palbociclib. (A) Representative images of cells stained with an amine reactive dye. (B) Cell volume was determined on a coulter counter. Error bars show standard deviation of three biological replicates. (C) DNA content determined by flow cytometry. (D and E) IMR90 cells were arrested in G1 with Palbociclib (1 μM) and grown in either 10% FBS or 0.2% FBS (starve). After 4 days, cell volume (D) was determined, Palbociclib removed, and EdU incorporation assayed 48 h thereafter (E). Asterisks indicate p < 0.05 (Student’s t test). (F–H) IMR90 cells expressing genetically encoded fluorescent nanoparticles (40 nm) were treated with Doxorubicin or Palbociclib and diffusion rates determined. (G, H) Diffusion coefficients in treated samples were significantly different from the controls (p < 5^∗^10^−120^, Kolmogorov-Smirnov Test, Error bars show SEM).

Increased cell size is a conserved feature of replicative senescence. Primary human cell lines increase in cell size when they enter a permanent cell-cycle arrest and senesce. Increased cell size is also associated with cellular senescence *in vivo* (Biran et al., 2017). Our observations in large yeast cells prompted us to investigate whether the cell size increase that occurs during senescence in human fibroblasts contributes to physiological changes and permanent cell-cycle block that characterizes cellular senescence. We induced senescence in primary human fibroblasts (IMR90) by treating cells with the DNA damaging agent Doxorubicin for 24 h. Within 9 days after Doxorubicin treatment, cell size increased from 1.9 pL to 16 pL (Figures 7A and 7B). This 8-fold increase in cell volume was accompanied by only a small (20%) increase in cells with 4N or greater ploidy (Figure 7C). Cells induced to undergo senescence using the Cdk4/6 inhibitor Palbociclib showed a 7-fold increase in cell size after 10 days without a corresponding increase in DNA content (Figures 7B and 7C). Cell senescence is therefore accompanied by a large decrease in DNA:cytoplasm ratio.

To determine whether large cell size contributes to the permanent cell-cycle arrest that is a hallmark of senescence, we treated cells with Palbociclib for 4 days, removed the drug and then assessed proliferative potential by measuring EdU incorporation. During the 4-day arrest, cell size increased by 2-fold and only 5% of cells were able to re-enter cell division after removal of the drug (Figures 7D and 7E). Strikingly, when we prevented cell growth during the Palbociclib treatment by reducing the serum concentration in the medium, proliferative potential was restored to wild-type levels (Figures 7D and 7E). This is in agreement with previous observations showing that p21 induced cellular senescence can be prevented by restricting cell growth (Demidenko and Blagosklonny, 2008). We conclude that increased cell size interferes with cell proliferation in mammalian cells and propose that it contributes to the permanent cell-cycle arrest that is associated with cellular senescence.

Could, like in yeast, cytoplasm dilution be responsible for the defects observed in oversize IMR90 cells? Because large IMR90 cells became very fragile when detached from substrate, we were not able to directly assess cellular protein and RNA levels. Instead, we determined the degree of macromolecular crowding by measuring diffusion rates of genetically encoded multimeric nanoparticles (GEMs; Delarue et al., 2018). Diffusion of 40 nm sized GEMs is determined by the concentration of ribosomes in the cytoplasm (Delarue et al., 2018). We found that GEM diffusion rates increased by 30% in fibroblasts treated with Doxorubicin or Palbociclib (Figures 7F–7H). This observation indicates that cytoplasm dilution occurs in large cells. Because starvation also decreases macromolecular crowding (Delarue et al., 2018) we did not examine GEM diffusion in cells that were starved during Palbociclib treatment. We conclude that large cell size and a low DNA:cytoplasm ratio contribute to the physiological changes and permanent cell-cycle arrest that accompany mammalian cellular senescence.

## Discussion

In this study we determined why maintenance of a cell type-specific DNA:cytoplasm ratio is critical for cell function. DNA copy number has previously been shown to become limiting for RNA and protein synthesis during prolonged cell-cycle arrests in *S. pombe* (Zhurinsky et al., 2010). Our work indicates that this does not automatically lead to attenuation in cell volume increase, but instead, cells continue to expand in size leading to dilution of the cytoplasm. Our data further suggest that this cytoplasm dilution contributes to loss of cell function during senescence. The uncoupling of RNA and protein synthesis from cell volume is surprising and indicates that these two processes are regulated independently. Determining how cell volume increase and RNA and protein synthesis are normally coordinated with each other and how this coordination is lost during prolonged cell-cycle arrests remains to be determined.

### How Does DNA Limit RNA and Protein Synthesis?

A central question that arises from our study is how DNA becomes limiting in large cells and why this leads to a coordinated decrease in gene expression of all genes. One possibility is that a universal maximal transcription limit exists. For example, the number of RNA polymerases transcribing a gene at the same time could be limited by gene length. In such a scenario, highly expressed genes would encounter such a universal maximal transcription limit at smaller sizes than weakly expressed genes, resulting in a global imbalance of gene expression. Our gene expression analysis argues otherwise: both poorly and highly expressed genes fail to scale with cell volume beyond 200 fL. Instead, expression of genes critical for transcription and translation declines as part of a gene expression program known as the environmental stress response (ESR). ESR activation may help to downregulate gene expression in a coordinated fashion. Whether low DNA:cytoplasm ratio itself activates the ESR and to what extent ESR activation contributes to the coordinated transcription attenuation remains to be determined.

### The Consequences of RNA and Protein Dilution

The effects of protein and RNA dilution on cell physiology are profound. Theoretical considerations predict that unstable proteins are more rapidly diluted than stable proteins as cell volume increases and protein synthesis stops scaling. As cell division and gene expression regulation rely on threshold concentrations of unstable proteins, this property of cytoplasm dilution is a plausible explanation for why these processes fail in oversized cells. Not only are individual proteins diluted, but because the general transcription and translation machineries are especially affected by cytoplasm dilution, the capacity to make new proteins is also reduced. This makes gene induction less efficient and has profound consequences for processes that heavily rely on *de novo* gene expression such as cell-cycle progression or adaptation to altered environmental conditions. However, the effects of decreasing global protein and RNA concentration is likely to have much broader effects on cell physiology. Dilution alters biochemical reaction rates and basic biophysical properties of the cytoplasm such as viscosity, diffusion rates and macromolecular crowding (Delarue et al., 2018, Zhou et al., 2008). Considering the broad impact of cytoplasmic dilution, even small changes in global protein concentration could add up to big effects.

### Decreased DNA:Cytoplasm Ratio-a Characteristic of Senescence

We have identified one physiological context where budding yeast and human cells reach sizes where cytoplasm dilution could begin to take place: senescence. In yeast, many factors such as accumulation of extra chromosomal rDNA circles, changes in vacuolar pH, accumulation of damaged proteins and protein aggregates have been proposed to contribute to the functional decline of old cells. Our results suggest that increased cell size is an additional contributor to replicative aging in yeast: Increasing cell size is sufficient to shorten lifespan. We have also observed that cell density decreases as old cells grow larger than 200 fL, raising the possibility that the defects associated with old age could be a consequence of cytoplasm dilution. We note, however, that the decrease in cell density could also be due to expansion of the vacuole, which has been observed in old cells (Lee et al., 2012). Perhaps old cells actively increase vacuolar volume to counteract dilution of the cytoplasm. Plant cells use this strategy to generate large cells without the need to synthesize large amounts of cytoplasm.

Our results suggest that increased cell size also contributes to senescence in mammalian cells. Artificially increasing cell size by maintaining cells arrested in G1 is sufficient to inhibit cell proliferation once cells have been released from the G1 block. Furthermore, the dramatic cell size increase observed in senescent human fibroblasts is accompanied by a decrease in macromolecular crowding. These results indicate that in humans too, DNA becomes limiting for cell function during cellular senescence. We propose that because cell division and cell growth are only loosely entrained, stochastic cell-cycle arrests result in cell size increase which in turn promotes senescence.

In summary, our results indicate that in eukaryotes, maintenance of a cell type specific DNA:cytoplasm ratio is critical for most, perhaps all cellular functions. The size range that supports optimal cell function is dictated by DNA copy number. Surpassing the upper size limit leads to pathologies and aging. Alterations in cell size therefore need to be taken into account in future studies that aim to understand changes in cell physiology during differentiation and cellular senescence.

## STAR★Methods

### Key Resources Table

**Table:** 

REAGENT or RESOURCE	SOURCE	IDENTIFIER
**Antibodies**		

Mouse monoclonal αPgk1 (WB, 1:50,000)	Invitrogen	Cat# A6457; RRID: AB_2313773
Rabbit polyclonal αKar2 (WB, 1:200,000)	Gift from Mark Rose	N/A
Rabbit polyclonal αCdc28 (WB, 1:1,000)	Gift from Kim Nasmyth	N/A
Goat polyclonal αFus3 (WB, 1:1,000)	Santa Cruz Biotechnology	Cat# sc-6773; RRID: AB_671991
Rabbit monoclonal phospho-p44/42 MAPK (P-Fus3, WB, 1:1,000)	Cell Signaling Technology	Cat# 4370P; RRID: AB_2315112
Mouse monoclonal αV5	Life Technologies	Cat# R960-25; RRID: AB_2556564
Mouse purified polyclonal αRpb3	Bio Legend	Cat# 665004; RRID: AB_2565221
Rabbit polyclonal αTbp (Spt15)	Gift from S. Buratowski	N/A
HRP-αMouse (1:10,000)	GE	Cat# NA9310; RRID: AB_772193
HRP-αRabbit (1:10,000)	GE	Cat# NA934; RRID: AB_772206
HRP-αGoat (1:10,000)	abcam	Cat# Ab68851; RRID: AB_2199023
GAmmaBind G Sepharose	Amersham Biosciences	Cat# 17-0885-01

**Chemicals, Peptides, and Recombinant Proteins**		

Phleomycin	Sigma	Cat# P9564
Benomyl	Sigma	Cat# 381586
Thiolutin	CMS Chemicals	Cat# FT02783
Rapamycin	Sigma	Cat# R0395
Doxorubicin	Sigma-Aldrich	Cat# D1515
Palbociclib (PD-0332991)	Selleckchem	Cat# S1116
Protease Inhibitor	Thermo Fisher Scientific	Cat# 78429
Concanavalin A	MP Biomedicals	Cat# 2150710
EZ-Link Sulfo-NHS-LC-Biotin	Thermo Fisher Scientific	Cat# 21335
Streptavidin, Alexa Fluor 568	Molecular Probes	Cat# S11226
Wheat Germ Agglutinin (WGA), Alexa Fluor 488	Invitrogen	Cat# W11261
SYTOX Green Nucleic Acid Stain	Molecular Probes	Cat# S7020
Alexa Fluor 488 NHS Ester (Succinimidyl Ester)	Molecular Probes	Cat# A20000)
TMT11-plex tandem mass tag	Thermo Fisher Scientific	Cat# A34808
a-factor yeast mating pheromone	The Koch Institute Swanson Biotechnology Center – Biopolymers Core Facility	N/A
Pronase	Roche	Cat# 10165921001
Zymolyase	MP Biomedicals	Cat# 8320932
SuperScript III First-Strand Synthesis	Invitrogen	Cat# 18080-400
SYBR Premix Ex Taq	Clontech	Cat# TAKRR420A
Ribonuclease A	Sigma-Aldrich	Cat# R4642
OptiPrep Density Gradient Medium	Sigma	Cat# D1556

**Critical Commercial Assays**		

Quick Amp Labeling Kit, two-color	Agilent	Cat# 5190-0444
Yeast Expression Microarray (8x15k)	Agilent	Cat# G4813A
RNeasy Mini Kit	QIAGEN	Cat# 74104
Qubit RNA BR Assay Kit	Thermo Fisher Scientific	Cat# Q33210
Qubit dsDNA BR Assay Kit	Thermo Fisher Scientific	Cat# Q32850
Qubit 3 Fluorometer	Thermo Fisher Scientific	N/A
Imperial Protein Stain (Comassie)	Thermo Fisher Scientific	Cat# 24615
Bradford protein assay	Biorad	Cat# 5000006
Anti-Biotin MicroBeads	Miltenyi Biotec	Cat#130-090-485
LS Columns	Miltenyi Biotec	Cat#130-042-401
Pre-Separation Filters (30 mm)	Miltenyi Biotec	Cat#130-041-407
Click-iT EdU Alexa Fluor 488 Imaging Kit	Invitrogen	Cat# C10337

**Deposited Data**		

Microarray data (Table S1) and RNA sequencing data (Table S2)	This paper	GEO: GSE110704
*S. cerevisiae* reference genome (Saccer3)	Genome Browser, UCSC	http://hgdownload.cse.ucsc.edu/goldenPath/sacCer3/bigZips/
*C. albicans* reference genome (SC5314_A22)	Candida Genome Database	www.candidagenome.org

**Experimental Models: Organisms/Strains**		

All *Saccharomyces cerevisiae* strains used in this study are of the W303 strain background and are listed in Table S4	Amon Lab	N/A
*Candida albicans*	Gerald A. Fink Lab	SC5314
Primary human lung fibroblasts, IMR90	ATCC	Cat# CCL-186
IMR90 + PfV GEMs (pLH1396)	Holt Lab	N/A
HEK293T cells	Holt Lab	N/A

**Oligonucleotides**		

qPCR Primers Telomere IV: f-GCGTAACAAAGCCATAATGCCTCC, r-CTCGTTAGGATCACGTTCGAATCC	This paper	N/A
qPCR Primers *GAL1* UAS: f-ACGCTTAACTGCTCATTGCT, r-ACGCACGGAGGAGAGTCTT	This paper	N/A
qPCR Primers *GAL1* Promoter (TATA box): f-TTTTTAGCCTTATTTCTGGGGTAA, r-GTGGTTATGCAGCTTTTCCAT	This paper	N/A
qPCR Primers *GAL1* ORF: f-GCGCAAAGGAATTACCAAGA, r-TACCAGGCGATCTAGCAACA	This paper	N/A

**Recombinant DNA**		

Plasmid: pFA6a-TRP1-PGal1-3HA-CDC28	Amon Lab	p2294
Plasmid: *pFA6a-TRP1-PGAL1-turboGFP*	Amon Lab	p2432
Plasmid: URA3:GAL80pr-3V5-GAL80	Amon Lab	p2710
Plasmid: pEF1Alpha-Pfv-GS-Sapphire	Holt Lab	pLH1396

**Software and Algorithms**		

FIJI (ImageJ)	NIH	https://fiji.sc/
STAR version 2.5.3.a	(Dobin et al., 2013)	https://code.google.com/archive/p/rna-star/
GSEA 3.0	Broad Institute	http://software.broadinstitute.org/gsea/index.jsp
Gene Pattern ssGSEA Projection Module (V9)	Broad Institute	http://software.broadinstitute.org/cancer/software/genepattern
StarSearch software	Arjun Raj laboratory, University of Pennsylvania	http://rajlab.seas.upenn.edu/StarSearch/launch.html
Volocity (6.3)	Perkin Elmer	
MOSAIC for ImageJ	(Shivanandan et al., 2013)	https://bmcbioinformatics.biomedcentral.com/articles/10.1186/1471-2105-14-349
Matlab2018a	Mathworks, Inc. (2018).	https://www.mathworks.com/products/matlab.html
Nikon Elements	Nikon Instruments, Inc. (2017)	https://www.microscope.healthcare.nikon.com/products/software

**Other**		

Mini Bead Beater	Biosspec Products	N/A
FastPrep-24 MP	Biomedicals	N/A
Nunc Lab-Tek II Chambered Coverglass	Nunc	Cat# 155409
Multisizer 3 Coulter Counter	Beckmann Coulter	N/A
Elutriator: Avanti J-26 XP Centrifuge (Rotor JE 5.0)	Beckmann Coulter	N/A
Light Cycler 480 II	Roche	N/A
Bioruptor Waterbath Sonicator	Diagenode	N/A

### Contact for Reagent and Resource Sharing

Further information and requests for resources and reagents should be directed to and will be fulfilled by the Lead Contact, Angelika Amon (angelika@mit.edu)

### Experimental Model and Subject Details

Primary female human lung fibroblasts (IMR90, ATCC) were cultured in growth medium composed of DMEM (Invitrogen) supplemented with 10% FBS, 2 mM L-glutamine and 100 U/mL penicillin/streptomycin. IMR90 cells were used from passage one to fifteen.

*S. cerevisiae* strains are derivatives of W303. The strains used in this study and their genotypes are listed in Table S4. Cells were grown in yeast extract/peptone (YEP) supplemented with adenine (0.055 mg/mL) and tryptophan (0.8 mg/mL) and either 2% glucose (YEPD), 2% raffinose (YEPR) or 2% raffinose and 1% galactose (YEP R/G). Alternatively cells were grown in synthetic complete medium supplemented with 2% glucose (SCD) or 2% raffinose and 1% galactose (SC R/G). Yeast strains used in the presented experiments are as follows:

Figure 1

(A) A17896; (B) A2587, A40116; (C-E) A40116; (F) A40111; (G) A40116, A40125, A40126; (H) A40116, A40540

Figure 2

(A-C) A40111; (D, E) A40139; (F) A19062; (G) A34481; (H) A2587, A40132, A36338

A40137, A40138; (I) A17132, A2587, A6719

Figure 3

(A, B) A17896; (C) A40130; (D) A17896, A40130; (E) A17896 (F) A40541, A40549; (G) A40541-40549; (H) A17896

Figure 4

(A-F) A17896

Figure 5

(A-C) A40129, A40134; (D, E) A40128, A40133; (F, G) A17896, A40112

Figure 6

(A) A40131; (B) A17896, A40131; (C) A40140; (D) A35824; (E, F) A37624; (G) A40120

Figure S1

(A-D) A40550

Figure S2

(A) A40111, A40117, A40118, A40119; (B) A17896, A40120; (C-E) A40116, A40122, A40123; (F-H) A40111, A40117, A40119; (I) A40121

Figure S3

(A) A35251, A35252; (B-D) A35252; (E) A40113; (H) A40114, A40115

Figure S4

(A) A40111; (B) A40139

Figure S5

(A) A17896; (B) A40130; (C) A17896, A40130; (D) A17896; (E, F) A17896; (G) A2587, A17132

Figure S6

(A) A17896; (B, C) A31566; (D) A40130; (E) A17896; (F) A40130 (RNA), A17896 (Protein); (G) A40130

Figure S7

(A) A17896, A40112; (B) A40551, A40552; (C-E) A40553; (F) A17896, A40112

### Method Details

#### Yeast Strain Generation

PCR based transformation was used to integrate epitope tags and fluorescent proteins as described previously (Janke et al., 2004, Longtine et al., 1998). Transformed strains were crossed and dissected by micromanipulation to obtain desired genotypes. All strains used in this study are listed in Table S4.

#### Plasmid Construction

*pFA6a-TRP1-PGal1-3HA-CDC28* (p2294) was generated by PCR amplification of *CDC28* from genomic DNA and restriction cloned into *pFA6a-TRP1-PGal1-3HA* (Janke et al., 2004, Longtine et al., 1998) using AscI/BamHI in frame with an N-terminal 3HA tag.

*pFA6a-TRP1-PGAL1-turboGFP* (p2432) was constructed by PCR amplification of turboGFP from *pGateway-TurboGFP* (Evrogen, cat#FP521) and restriction cloning into *pFA6a-TRP1-PGAL1-GFP* (Longtine et al., 1998) using AscI/PacI.

*URA3:GAL80pr:3V5* (p2710) was generated by PCR amplification of the 1 kb sequence upstream of the *GAL80* start codon from genomic DNA. Gibson assembly was used to integrate the PCR fragment 5′ of the 3V5 epitope tag in plasmid pMH9 (3V5:URA/KAN). The resulting plasmid was used as a template for PCR based transformation replacing the upstream 1 kb sequence of the endogenous *GAL80* locus. Cloned plasmids were confirmed by sanger-sequencing. All plasmids used in this study are listed in the key resources table.

#### Yeast culture conditions

##### Cell-cycle arrest and release of yeast cultures

For G1 arrests, cells carrying the temperature sensitive *cdc28-4* or *cdc28-13* alleles were grown at 26°C overnight in YEPD or YEPR to an optical density (OD_600_) < 1, diluted to OD_600_ = 0.1 in fresh medium for 90 min before cells were shifted to the restrictive temperature (35°C for *cdc28-4*, 37°C for *cdc28-13*). For release from the cell-cycle arrest, cells were collected by centrifugation and all cells were re-suspended in fresh medium for further analysis.

*bar1Δ* (*bni1Δ* or *BNI1*) mutant cells were arrested in G1 in YEPD or YEPR by addition of 2 μg/mL alpha factor. For release from the alpha factor induced G1 arrest, cells were washed 1 time with an equal volume of culture medium and released into medium containing 25 μg/mL pronase (an enzyme that degrades alpha factor). Cell growth rate during the G1 arrest was slowed by (1) addition of 100 ng/mL cycloheximide or (2) replacing the growth medium with YEP/0.1% glucose when cells were shifted to 37°C.

For cell proliferation assays on plates, 10-fold serial dilutions of cells were spotted onto YEPD plates containing Thiolutin (2-4 μg/mL) and grown at permissive temperature (25°C).

##### Elutriation and cell-cycle arrest

For isolation of newborn daughter cells by centrifugal elutriation, cells were grown in 1 L YEPD at 30°C to OD_600_ = 3. Subsequently cells were collected by centrifugation, re-suspended in chilled YEP (no sugar) and briefly sonicated using a tip sonicator to break up clumps. Centrifugal elutriation was performed in YEP (no sugar) at 4°C. Cells were loaded into a pre-equilibrated Beckman elutriation rotor JE 5.0 at 2400 rpm at a flow rate of 10 mL/min. Loaded cells were allowed to equilibrate for 30 min. Flow rate was then gradually increased until small unbudded daughter cells started exiting the elutriation chamber, which occurred at a flow rate of 16-18 mL/min. Up to 1 L of cells was collected. Cells were concentrated by centrifugation, re-suspended in 25 mL chilled YEPD and placed in a 37°C shaker. After 30 min at 37°C, the culture density was adjusted to OD_600_ = 0.3 with pre-warmed YEPD medium, except for the experiment shown in Figure S6D, E where cells were diluted to OD_600_ = 0.075, 0.3 and 0.6 to assess the effect of cell density on growth rate and stress response induction.

##### Inducing genome duplication

To prevent cell division following S phase, checkpoint deficient cells were treated with Nocodazole: *cdc28-13 mad1Δ bub2Δ* mutant cells were grown in YEPD and arrested in G1 using alpha factor pheromone (5 μg/mL) for 2 h at 25°C. Subsequently, alpha factor was washed out and cells were released at 25°C into medium lacking pheromone. 60 min after alpha factor washout, Nocodazole (20 μg/mL) or DMSO were added to the culture and 75 min after pheromone removal, cultures were shifted to 37°C to arrest cells in the subsequent G1 phase. Nocodazole was removed 2.5 h after the alpha factor washout. To assess induction of *GAL1*, cells were treated identically except for that cells were grown in YEPR and that Nocodazole was washed out later (3.2 h after alpha factor washout).

##### Induction of the galactose and pheromone response

For *GAL1* promoter inductions, cells were grown to exponential phase in YEPR. The promoter was always induced at room temperature in YEP R/G (RT-qPCR, ChIP analysis, single molecule RNA FISH) or SC R/G (for time lapse microscopy).

The pheromone response was induced by exposing cells to 20 μg/mL alpha factor. For analysis of *FIG1* mRNA by RT-qPCR, cells were shifted to room temperature, collected by centrifugation and frozen in liquid nitrogen. For preparation of RNA for microarray analysis, arrested cells were filtered and re-suspended in fresh, pre-warmed medium (35°C) containing alpha factor and cells were collected after 40 min. For Fus3 phosphorylation analysis by western blot analysis, alpha factor was added directly to the arrested cultures without a prior shift to room temperature or removal of cycloheximide. Samples were taken immediately before and 5-, 15- and 30 min after alpha factor addition.

##### Pedigree Analysis

*GPD1pr-cdc28-13* cells were grown to exponential phase and arrested at 37°C. Cells were transferred to a YEPD/agar plate (2% glucose) and 50 unbudded cells per condition were aligned using a micromanipulator microscope. Cells were incubated at 25°C and daughter cells were regularly removed from mother cells and number of daughters produced per mother was counted. Plates were stored at 4°C for up to 10 h during the experiment. Only cells that completed at least one cell division were included in the analysis.

##### Isolation and analysis of aged cells

Aged cells were purified as described in (Smeal et al., 1996). Cells from glycerol stocks (−80°C) were plated onto YEPG (2% glycerol) agar plates for 24 h at 30°C and transferred onto YEPD plates for another 24 h. Subsequently, cells were grown to exponential phase in YEPD medium containing 100 μg/mL ampicillin. Roughly 1.5 × 10^8^ cells were harvested by centrifugation and washed with chilled PBS/pH8. Cells were labeled with EZ-Link Sulfo-NHS-LC-Biotin (8 mg/mL) for 30 min at 4°C. Subsequently cells were washed with PBS pH 8.0/100 mM glycine to remove excess biotin. Biotinylated cells were grown at 30°C for 4 h (young) and12 h (middle aged) in YEPD. For purification of labeled mother cells, harvested cells were incubated with magnetic anti-biotin micro beads in PBS/1%BSA for 15 min at 4°C and washed in the same buffer. Next, labeled cells were purified using LS depletion magnetic columns following the manufacturer’s instructions. To obtain cells that had undergone more than 8 generations, the sorted cells were grown for an additional 6-8 divisions after which the cell sorting was repeated.

Cell volume (coulter counter) and density measurements (SMR) were performed immediately after the isolation of old cells. An aliquot of old cells was used to determine mean cell age. Unlabeled young cells grown in the same culture as the aged cells (cells that did not bind to the magnetic column) served as a control for the cell volume measurement (Figure 6A). For induction of the *GAL1* promoter in aged cells, cells were grown in YEPD (2% glucose), biotin labeled, aged for 8 generations in YEPD, sorted and then inoculated in YEPR (2% raffinose) for 14 h. After this period, a second purification was performed and *GAL1* expression was induced with 1% galactose. *GAL1* induction was determined by microscopy (*GAL1pr-GFP*) and by single molecule RNA FISH. For single molecule RNA FISH, calcofluor was used to identify cells with multiple bud scars (old cells).

To assay the induction of the pheromone responsive *FIG1*-GFP reporter, isolated old cells were re-suspended in fresh YEPD medium and grown for 2 h at 30°C before 20 μg/mL pheromone was added to the culture. Cells were fixed at different times in 4% formaldehyde for 5 min, and subsequently washed with PBS/100 mM glycine. FACS was used to identify aged cells (Biotin+) and the degree of GFP induction. Biotin negative cells that were co-purified with the aged cells, as well as young biotin labeled cells, that were isolated 4 h after the initial biotin labeling, served as controls.

#### Mammalian cell culture conditions

##### Senescence induction

To induce senescence, cells were treated with Doxorubicin (100 ng/mL) for 24 h. To arrest cells in G1 cells were grown in the presence of Palbociclib (1 μM). Arrested cells were trypsinized and split 3 days after treatment and medium was replaced regularly. For analysis of GEM diffusion rates, IMR90 cells expressing 40 nm-GEM nanoparticles were treated for 24 h with 100 ng/mL and 200 ng/mL Doxorubicin, and 5 μM Palbociclib and medium was replaced regularly.

##### Proliferation assay

To assess proliferative capacity after prolonged G1 arrest, IMR90 cells were grown to 80% confluence. Subsequently, Palbociclib (1 μM) was added and cells were grown in either the presence of 10% or 0.2% FBS (starvation medium). After 2 days, Nocodazole (200 μM) was added to the Palbociclib treated cells to remove cells that had failed to arrest in G1 by mitotic shake off. Vehicle was added to the control cells. 4 days after Palbociclib addition, cells were washed twice and released into growth medium supplemented with 10 μM EdU for another 2 days. EdU incorporation was visualized using the Click-iT EdU Alexa Fluor 488 Imaging Kit (Invitrogen, C10337) following the manufacturer’s instructions. DNA was stained with Hoechst.

##### Virus production and cell transduction

To produce lentivirus, 800,000 HEK293T cells were plated in 10 mL medium in 15 cm dishes. The next day, each well was transfected with 24 μg vector, 1.2 μg tat, 1.2 μg rev, 1.2 μg gag/pol, and 2.4 μg of vsv-g DNA with 90 μL trans-IT in 2 mL DMEM. Supernatants were collected at 24 h and 48 h after transfection and spun down at 300 rcf to pellet cells. Viral supernatants were then concentrated using 30 kDa EMD Millipore Amicon Ultra-15 Centrifugal Filter Units and stored at −80°C until use. Stable IMR90 cell lines were created by lentiviral transduction with pLH1396. In order to transduce these cell lines, 50,000 cells were plated in 2 mL of medium in 6 well plates. The next day, medium was removed and replaced with medium containing 8 μg/mL polybrene. 2 μL of concentrated virus was added to the well and then the media was replaced after 24 h.

#### Cell staining

Purified, Biotin-labeled aged yeast cells were stained with fluorophore-coupled Streptavidin to identify old cells on the microscope and during flow cytometry (FACS) analysis. Wheat germ agglutinin (WGA) was used to stain bud scars to determine replicative age. Both dyes were used at a 1:1000 dilution in synthetic complete medium (live cells) or PBS (fixed cells) for 15 min.

For staining of total cellular protein in yeast, cells were fixed for 5 min in 4% formaldehyde, washed 2x with PBS containing 100 mM glycine and permeabilized in 70% ethanol at −20°C. Subsequently, cells were washed 1x with 0.2M Sodium Bicarbonate. An equivalent of 0.15 OD_600_ units of cells were used for staining in 0.5 mL 0.2M sodium bicarbonate containing 50 μg/mL Alexa Fluor 488 NHS Ester (Succinimidyl Ester) for 30 min at room temperature.

Human fibroblasts grown on a coverslip were fixed for 10 min in 4% paraformaldehyde, washed with PBS and permeabilized in 100% methanol for 10 min at −20°C. Methanol was removed and cells washed once with 0.2M Sodium Bicarbonate, followed by staining in 0.5 mL 0.2M sodium bicarbonate containing 50 μg/mL Alexa Fluor 488 NHS Ester (Succinimidyl Ester) for 30 min at room temperature.

DNA staining for flow cytometry analysis was performed as follows: Yeast cells were fixed in 70% ethanol at 4°C. Cells were collected by centrifugation, resuspended and washed with 50 mM sodium citrate. Cells were then treated for 1 h with Ribonuclease A (250 μg/mL) at 37°C, after which cells were collected by centrifugation and resuspended in 1 mL 50 mM sodium citrate containing 1 μM SytoxGreen. Cells were briefly sonicated to break up clumps and analyzed by flow cytometry. Human fibroblast cells were dissociated from culture plates using trypsin and mixed with 100% chilled ethanol to a final concentration of 70%. Ethanol fixation was performed at −20°C for at least 30 min. Cells were then collected by centrifugation, washed with PBS and resuspended in 0.5 mL PBS containing 100 μg/mL propidium iodide and RibonucleaseA (2 mg/mL) and incubated for 1 h at 37°C prior to analysis by flow cytometry.

#### Analysis of Cell Volume and Density (SMR)

For cell volume analyses, cells were sonicated briefly with a tip sonicator and the volume of 50,000 cells was measured on a Beckman coulter counter. The volume and density of single cells were measured using a suspended microchannel resonator (Bryan et al., 2010, Son et al., 2015). The SMR device was fabricated at CEA-LETI, Grenoble, France, with a geometry and dimensions identical to those in (Son et al., 2015). The device was vibrated using a piezo-ceramic plate in order to resonate the SMR’s cantilever in the second flexural vibration mode. The vibration of the cantilever was monitored with piezoresistors and the system operation temperature was controlled by placing the SMR on top of a heat controlled copper plate. As the vibration frequency of the cantilever is directly proportional to the mass of the cantilever, flowing a cell through a channel embedded within the cantilever allows for the quantification of the cell’s buoyant mass. Once the cell passes to the other side of the cantilever, it is transferred to more dense medium containing 50% OptiPrep (Sigma-Aldrich). Reversing the flow and analyzing the same cell in the more dense medium provides the cell’s buoyant mass in two media of different density, from which absolute mass, volume and density were derived. Elutriated *cdc28-13* cells were placed in a 37°C incubation chamber connected to this SMR setup and individual G1-arrested cells were analyzed over the course of a 7.5 h period. To measure the density of aged cells, purified old cells were sonicated and kept in a 30°C incubation chamber and individual cells were analyzed for a duration of 2 h.

#### Microscopy

Time lapse experiments, bud scar images of aged yeast cells and EdU incorporation into IMR90 cells were imaged using a DeltaVision Elite microscope platform (GE Healthcare Bio-Sciences) using the 60X Plan APO 1.42NA objective and a CoolSNAP HQ2 camera. The microscope has an environmental control chamber.

For single molecule RNA FISH imaging a Nikon TI-E imaging microscope with a 100x oil immersion objective (NA 1.4) and an ORCA-FLASH 4.0 camera (Hamamatsu) and NIS-element software (Nikon) was used.

To measure cell volume using PGK1-mCherry and for measurements of the concentration of mCherry fusion proteins (Figures 3F and 3G) and for single particle tracking in IMR90 cells we used an Andor Yokogawa CSU-X confocal spinning disc on a Nikon TI Eclipse microscope and fluorescence was recorded with a sCMOS Prime95B camera (Photometrics) with a 60x (yeast) or 100x (IMR90) objective.

For live cell microscopy, cells were collected by centrifugation (800 g/2 min), re-suspended in synthetic complete medium and mildly sonicated to break up cell clumps. Cells were plated in 8-well LabTek Chambers, which were pre-incubated with 2 mg/mL ConA for ≥ 10 min and washed 3x with medium. Cells were added and allowed to settle for 1-5 min before unbound cells were washed away with fresh medium. The chamber was subsequently filled with 0.5 mL medium and placed on the microscope stage. To minimize light exposure, images were acquired using 2x2 or 4x4 binning. Stacks of 10 images (Δz = 1.5 μm) were acquired every 5 min.

#### Quantitative Transcriptome Analysis

##### RNA Isolation and quantification

For quantification of total cellular RNA and RT-qPCR analysis 2 mL of a cell suspension (∼10^7^ cells) were collected by centrifugation (30 s/10,000xg) and snap-frozen in liquid nitrogen. Pellets were re-suspended in 400 μL TES buffer (10 mM Tris pH7.5, 10 mM EDTA, 0.5%SDS), 400 μL acid phenol and 100 μL glass beads were added and the mixture was incubated for 45 min in a thermoshaker (900 rpm) at 65°C. After centrifugation, 350 μL of the aqueous phase were mixed with 1 mL ethanol and 40 μL 3M sodium acetate and RNA was precipitated overnight at 4°C. The precipitated RNA was pelleted by centrifugation, air-dried and re-suspended in water and subjected to DNase treatment and column purification (RNAeasy, QIAGEN). Total cellular RNA was quantified either after ethanol precipitation using the Qubit RNA BR assay or after column purification using a spectrophotometer (NanoDrop).

##### RT-qPCR analysis

800 ng of purified RNA and random hexamer primers were used for the reverse transcription reaction (Superscript, QIAGEN). *FIG1* mRNA and *GAL1* mRNA levels relative to *ACT1* mRNA were quantified by quantitative PCR (Sybr Green, Roche Light Cycler). The primers and primer sequences used in RT-qPCR reactions are listed in the key resources table.

##### Microarray analysis

For microarray analysis, *cdc28-4* and *CDC28* cells were arrested in G1 at 35°C for 2 h or 6 h in YEPD (±100 ng/mL cycloheximide). Cells were then collected and washed to remove cycloheximide and transferred into fresh, prewarmed YEPD (35°C) with or without 20 μg/mL alpha factor and incubated for 40 min at 35°C. Cells were harvested by filtration and snap frozen in liquid nitrogen. RNA was purified as described above, and RNA quality determined using a Bioanalyzer (Agilent). Agilent Quick Amp Labeling Kit (two-color) was used to label and amplify the purified RNA per the manufacturer’s instructions. 500 ng of total RNA was used as input for the labeling reaction. RNA isolated from *cdc28-4* cells arrested for 2 h served as reference RNA for all samples. 300 ng of labeled sample and reference RNA, each containing more than 2.5 pmol dye, were mixed, fragmented (60°C for 30 min) and hybridized (65°C for 18 h) to a yeast expression microarray (Agilent). Microarrays were analyzed on an Agilent Scanner.

##### RNA Seq analysis

For total RNA sequencing, an equal number (∼10^7^) of *S. cerevisiae* cells of different sizes were mixed with a constant number (∼1.5x 10^6^) of exponentially growing *C. albicans* cells. RNA was purified as described above. RNA quality was determined using a Fragment Analyzer (Advanced Analytical) and 300 ng of total RNA were used to prepare cDNA using the Illumina TruSeq kit, skipping the mRNA isolation step. Illumina libraries were then prepared from the cDNA and indexed using NexteraXT (Illumina) and sequenced on an Illumina HiSeq2000 (50 nt single end read).

##### Single molecule RNA-FISH analysis

Single molecule RNA FISH was performed as described previously (van Werven et al., 2012). Cells were fixed with 4% formaldehyde overnight at 4°C and then treated with 50 mg/mL zymolyase for 15 min at 30°C. Digested cells were collected and stored in 80% ethanol. Subsequently, cells were hybridized with fluorophore labeled probes from Biosearch Technologies (Stellaris custom FISH probes) directed to *GAL1* (AF594) and the internal control *ACT1* (Cy5). Images were collected in DIC, GFP, DAPI (calcofluor), AF594 (*GAL1*), Cy5 (*ACT1*) channels, and slices were spaced by 0.3 μm. ImageJ software was used to make maximum intensity Z projections of the images. Subsequently, StarSearch software (Raj laboratory, University of Pennsylvania) was used to determine number of transcripts in single cells. Comparable thresholds were used to count the RNA foci in single cells. Only cells positive for the internal control *ACT1* were used for the analysis.

#### Protein Extraction and Analysis

##### Western Blot analysis

For western blot analysis, 1 mL of cell suspension (∼0.5x10^7^ cells) was mixed with 100 μL of 50% trichloric acid (TCA) and incubated for > 10 min at 4°C. Cells were then collected by centrifugation (30 s/10,000 g), re-suspended in acetone and pelleted by centrifugation. The pellet was air-dried, mixed with 100 μL breakage buffer (50 mM Tris-Cl at pH 7.5, 1 mM EDTA, 2.75 mM DTT, 1x protease inhibitor cocktail) together with 100 μL glass beads and cells were broken by vigorous shaking on a mini-beadbeater (5 min). Subsequently, the lysate was mixed with 50 μL 3x SDS-Sample Buffer (187.5 mM Tris oh6.8, 6% β-mercaptoethanol, 30% glycerol, 9% SDS, 0.05% bromophenol blue), boiled for 10 min and the lysate was cleared by centrifugation (2 min/21000xg). Between 1.5 μL and 20 μL of lysate were separated on SDS-PAGE. For western blot analysis proteins were transferred onto nitrocellulose membrane. The membrane was incubated with PBS (pH 7.4), 0.1% Tween-20, 1% Milk, 1% BSA for 30 min before the primary antibody was applied. Antibodies were diluted in (PBS (pH 7.4), 0.1% Tween-20, 1% Milk, 1% BSA, 0.1% NaN_3_). Antibodies and the corresponding dilution for western blot analysis are listed in the key resources table.

##### Quantification of extracted protein

To quantify the amount of total protein per cell, 1 mL of cells arrested in G1 were harvested at 1 h intervals after elutriation (initial OD_600_ = 0.3). Protein was extracted as described above, except that cells were lysed in 50 μL 8M Urea, 200 mM EPPS pH8.5, 1x protease inhibitor cocktail. Complete cell lysis was confirmed by microscopic examination of the extract. The lysates were mixed with 3x SDS sample buffer and boiled for 10 min before the lysate was cleared by centrifugation (2 min/21,000xg). 4 μL of lysate was separated on SDS-PAGE (15 well, 4%–12% gradient gel) and proteins were stained with comassie blue for 1 h and de-stained overnight in water. To detect possible effects of nonlinearity in the comassie staining, different sample volumes were analyzed for different time-points: 10 μL, 7.5 μL, 5 μL, 2.5 μL, 2.5 μL, 2.5 μL were loaded for the 1 h, 2 h, 3 h, 4 h, 5 h, 6 h arrest time-points, respectively. The results were almost identical (Figures 3C and S5C). For the quantification of total soluble protein per cell, cells were pelleted by centrifugation and snap frozen in liquid nitrogen. Cell pellets were re-suspended in (50 mM TrisHCl ph7.5, 150 mM NaCl, 1x protease inhibitor cocktail) and broken with glass beads on a bead beater (5 min). Complete cell lysis was confirmed by microscopic examination of the extract. Lysates were cleared by centrifugation (20 min/20,000xg) and protein concentration was measured using the Bradford protein assay.

#### Quantitative Proteomics

##### Protein Extraction, Digest, and TMT Labeling

Newborn *cdc28-13* daughter cells (G1) were isolated by centrifugal elutriation and arrested in G1 at 37°C. An equal number of cells (∼10^8^) was collected at different points during the G1 arrest, washed with PBS and pellets were snap frozen in liquid nitrogen. Three technical replicates for each time point were collected. Cells were lysed in 0.5 mL 8M Urea, 200 mM EPPS pH8.5, containing a complete mini protease inhibitor tablet (Roche). Lysis was performed at 4°C by 9 cycles of bead beating (1 mL ceramic beads, Biospec 11079105z) on a FastPrep, Cycles: 45 s, Level 6). Complete cell lysis was confirmed by microscopic examination of the extract. Proteins were separated from beads via centrifugation after piercing the bottom of the tube containing beads with a hot needle, and the lysate was cleared by centrifugation at 14,000xg for 10 min. This yielded 90 μg of protein for the 1 h arrested samples, 300 μg for the 3 h arrested samples, 390 μg for the 5 h arrested samples and 420 μg for the 7 h arrested samples. Samples were reduced with 5 mM TCEP (Sigma) for 25 min at room temperature, followed by alkylation of cysteine residues with 10 mM iodoacetamide (Sigma) for 30 min in the dark. Alkylation reactions were quenched by adding 5 mM DTT for 15 min in the dark. Extracts were purified by methanol-chloroform precipitation, dried in a vacuum centrifuge to near-dryness, then resuspended in 200 mM EPPS pH 8.5. Proteins were digested with Lys-C (Wako Chemicals) using a 100:1 protein to protease ratio overnight at room temperature, shaking in a vortex. Subsequently, the protein-peptide mixture was digested with trypsin (Promega) using a 100:1 protein to protease ratio for 6 h at 37°C. Following trypsin digest, samples were cooled to room temperature and anhydrous acetonitrile (ACN) was added to each sample to a final concentration of 30%. Isobaric labeling of peptides was performed using TMT11-plex tandem mass tag (TMT) reagents (Thermo Fisher Scientific). TMT reagents (5 mg) were dissolved in 256 μL anhydrous ACN and the amount of TMT reagent added to each sample was scaled according to peptide concentration. 8 μL TMT reagent was added to the ∼100 μg 1 h arrested sample, 24 μL added to the 3 h arrested samples, and 36 μL added to the 5 h and 7 h arrested samples. After 1 h of TMT labeling at 25°C, the reaction was quenched by adding hydroxylamine (Sigma) at a final concentration of 0.5%, and samples were incubated for an additional 15 min at 25°C. Sample labeling was confirmed to be > 90% in all samples. Labeled peptides were combined, acidified with 20 μL formic acid, and dried via vacuum centrifugation. The near-dry labeled peptide sample was resuspended in 5%ACN/5% formic acid to ensure pH∼2, followed by desalting via C18 SPE Sep-Pak cartridges (200 mg). Samples were then dried via vacuum centrifugation.

##### Offline basic pH reversed-phase HPLC fractionation

Dried TMT labeled peptides were solubilized in buffer A (5% ACN, 10 mM ammonium bicarbonate, pH 8.0) and separated by an Agilent 300 Extend C18 column (3.5 μm particles, 4.6 mm ID x 220 mm in length). Using an Agilent 1260 binary pump coupled with a degasser and a single wavelength detector set at 220 nm, a 60-min linear gradient from 13% to 40% acetonitrile in 10 mM ammonium bicarbonate pH 8 (flow rate of 0.6 mL/min) separated the peptide mixtures into a total of 96 fractions. 96 Fractions were consolidated into 12 samples in a checkerboard manner, acidified with 10 μL of 20% formic acid and vacuum dried. Each sample was re-dissolved in 5% FA/5% ACN, desalted via StageTips, dried via vacuum centrifugation, and reconstituted for LC-MS/MS analysis.

##### Data Acquisition: Oribtrap Fusion Lumos Parameters

All MS analyses were performed on an Oribtrap Fusion Lumos (Thermo Fisher Scientific) coupled to a Proxeon nLC-1200 ultra-high-pressure liquid chromatography (UPLC) pump (Thermo Fisher Scientific). Peptides were separated onto a packed 100 μM inner diameter column ∼35 cm of Accucore resin (2.6 μm, 150Å, Thermo Fisher Scientific), and a linear gradient of Buffer A (97.4% H_2_O, 2.5% ACN, 0.1% FA) to Buffer B (97.4% ACN, 2.5% H_2_O, 0.1% FA), consisting of 2%–23% of Buffer B over 120 min at ∼500 nl/min was used for separation. Each analysis used the MultiNotch MS3-based TMT method (McAlister et al., 2014). For analysis with the Orbitrap Fusion Lumos mass spectrometer, the scan sequence began with an MS1 spectrum collected at 50,000 orbitrap resolution with an automatic gain control (AGC) target of 4x10^5^, max injection time of 50 ms, and mass range of 400-1500*m/z*, with centroid data collection. The 10 most intense ions were selected for MS2/MS3 analysis. MS2 analysis consisted of collision-induced dissociation (CID) with an AGC of 2x10^4^, normalized collision energy (NCE) 35%, maximum injection time 120 ms, and isolation window of 0.7Da. For MS3 acquisition, the precursors were fragmented by high energy collision-induced dissociation (HCD) and analyzed via the orbitrap (AGC 1x10^5^; NCE 65%, maximum injection time 150 ms; resolution was 50,000).

#### Chromatin Immunoprecipitation (ChIP) analysis

*CDC28* or *cdc28-13* mutant cells were grown or arrested at 37°C in YEPR (2% raffinose). 5x10^8^ cells per condition (25 OD_600_ units) were collected by centrifugation and re-suspended in 40 mL YEPR or YEP R/G and incubated for 30 or 60 min. For ChIP analysis, formaldehyde was added to the culture at a final concentration of 1% and cells were fixed for 20 min at room temperature. Glycine was added (400 mM) and the incubation continued for 5 min at room temperature. Cells were washed 1x with water and 1x with FA buffer (50 mM HEPES-KOH pH7.5, 150 mM NaCl, 1 mM EDTA, 1% Triton X-100, 0.1% Na Deoxycholate, 0.1% SDS). Cells were re-suspended in 1 mL FA buffer supplemented with extra SDS to reach a final concentration of 0.5%, and 1 mL ceramic beads. Cells were broken on a Fast Prep until > 80% of cells were lysed (5-10 cycles, Intensity 6.5, 45 s cycles). Ultracentrifugation (1 h/200,000x g) was used to pellet chromatin. Pellets were mechanically disrupted in 250 μL FA buffer (0.1% SDS) and transferred to 1.5 mL TPX Microctubes. Samples were sonicated in a Bioruptor Waterbath Sonicator (Diagenode) for 4x10 cycles (high intensity). The sonicated lysate was cleared by centrifugation at 4°C (20 min/20,000x g). For immuno precipitation, dsDNA concentration was quantified using a Qubit reader and 100 μg DNA was used per precipitation reaction with the volume adjusted to 1 mL using FA buffer. Additional NaCl was added to a final concentration of 275 mM (Input). 15 μL of prewashed G-protein coupled Sepharose beads were used per tube together with antibodies against V5 (1 μL), Rpb3 (RNA Pol II, 2 μL), Spt15 (TBP, 1 μL) and rotated overnight at 4°C. Beads were sequentially washed in FA buffer (275 mM NaCl), FA buffer (500 mM NaCl), TE plus detergents (10 mM Tris-HCl, pH8, 0.25M LiCl, 1 mM EDTA, 0.5% NP-40, 0.5% NaDeoxycholate) and 1x TE (10 mM Tris-HCl pH8, 1 mM EDTA). DNA was eluted from the beads using 180 μL elution buffer (50 mMTris pH8/1 mMEDTA/1% SDS) at 65°C. Volume of the IP and the input sample (50 μL) was brought to 400 μL using 1xTE (10 mM Tris-HCl, 1 mM EDTA) and crosslinks were reversed by incubation in 1 mg/mL pronase at 42°C for 1 h and subsequently for 5 h at 65°C. Fragments were purified by phenol:chloroform extraction, followed by a chloroform extraction and precipitated in 450 mM LiCl, 0.1 g/mL Glycogen and 75% ethanol over night at −20°C. Precipitated DNA was pelleted, air-dried, re-suspended in 1xTE and analyzed by quantitative PCR using Sybr Green on a Roche Light Cycler 480. qPCR primers to sequences were as follows: Telomere of Chromosome IV (Tel IV, negative control), *GAL1* upstream activating sequence (*GAL1* UAS, binding site of Gal4 and Gal80), *GAL1* promoter (TBP and RNA Pol II binding site), *GAL1* open reading frame (*GAL1* ORF). Primers and sequences used for quantitative PCR analyses are listed in the key resources table.

### Quantification and Statistical Analysis

#### Microscopy Image Analysis

For analysis of Whi5-tdTomato, Spc42-GFP, Rfa1-mCherry localization, maximal intensity projections were generated and movies were analyzed manually. To quantify *CLN2pr*-GFP and *CLB2pr*-GFP expression GFP intensity was measured on maximal projections as opposed to on an individual z stack to reduce the contribution of the vacuole. Maximal projection allows to “focus” on the cytoplasm. Cells were outlined manually based on DIC images and mean GFP signal intensity was measured using Volocity (Version 6.3, Perkin Elmer). The signal of every cell was corrected in a first step for mean background fluorescence, measured at each time point throughout the movie in 3 empty positions in each field. In a second step, cellular auto fluorescence was accounted for by subtracting the average GFP signal of the same cell from the first 3 frames of the movie (when cells are still arrested in G1 and GFP expression is repressed. Average induction rate was determined by linear regression during the period when the GFP signal increased linearly. Amplitudes were determined by subtracting minima from maxima. Note that none of the background corrections affected the GFP induction rates and amplitudes, which were used for statistical analysis, but it made data visualization more intuitive (Figures 2A and S4B).

#### Quantification and analysis of GEM diffusion

Tracking of GEM particles in IMR90 cells was performed using the Mosaic suite of FIJI, with the following typical parameters: radius = 3, cutoff = 0,15% of fluorescence intensity, a link range of 1, and a maximum displacement of 7 px.

Effective diffusion rates were extracted from the particle trajectories as described (Delarue et al., 2018): The time-averaged mean-square displacement (MSD) was determined as well as the ensemble-average of the time-averaged MSD. As measured previously (Delarue et al., 2018) the diffusion of the tracer particle is subdiffusive, and generally obeys the following law:

$$\mathrm{MSD}\left(\tau \right)=4K\tau^{\alpha }$$

where α is the power exponent of the anomalous diffusion, and α < 1 in the case of a subdiffusive behavior. In this case, the apparent diffusion coefficient, Κ, is not in units of μm^2^/s, but rather in units of μm^2^/s^α^.

Individual particle trajectories were characterized by calculating apparent diffusion coefficients by fitting MSD with a linear (diffusive) time dependence at short timescales (< 100 ms). To do this, we calculated the MSD and truncated it to the first 10 points, and fit it with the following linear relationship:

$$\mathrm{MS}D_{\mathrm{truncated}}\left(\tau \right)=4D_{\mathrm{eff}}\tau$$

where D_eff_ is the effective coefficient of diffusion of the tracer particle. The Kolmogorov-Smirnov statistical test was used to assess the statistical difference between distributions.

#### RNASeq data analysis

RNA Seq reads were aligned to a combined *S. cerevisiae* (Saccer3) and *C. albicans* (SC5314_A22, www.candidagenome.org) genomic target using STAR version 2.5.3.a and a merged annotation file consisting of the ensemble *S. cerevisiae* annotation and SC5314_A22 annotation. Gene expression was quantified using rsem version 1.3.0. *S. cerevisiae* reads were normalized to gene length and number of total *C. albicans* reads in the sample. The unit used in the graphs is fragments per kilo base per million *C. albicans* reads (fpkmCa). Only protein coding genes (total 6691 genes) with an expression value of > 2 ([fpkmCa], log2) were considered for further analysis (5499 genes). Weakly expressed genes with a noisy expression pattern were filtered out by excluding genes with a coefficient of variation (CV)>0.5 across all samples ([fpkmCa], log2). This filtering reduced the list of genes included in the analysis to 5332. We performed gene set enrichment analysis (Subramanian et al., 2005) comparing two combined RNA Seq pooled datasets. A pool encompassing the 2 h, 2.5 h and 3 h (2-3 h arrest) time points was compared to a dataset encompassing time points 4.5 h, 5 h and 6 h arrest (5-6 h arrest). Log2 ratios were used as metric to determine gene ranks. Gene set permutation analysis was used to predict false discovery rates (FDR). For analysis of the environmental stress response, single sample GSEA projections for ESR repressed and ESR induced genes were generated for each time point, using gene pattern software, and row-centered across all time points. Table S2 summarizes the RNA Seq results. The raw data were submitted to GEO: GSE110704.

#### Microarray data analysis

Microarrays were scanned on an Agilent Scanner and features were extracted using the default settings. Data analysis was performed on the log_2_ values of the sample/reference signal ratio. Genes that were induced more than 4-fold in wild-type cells treated with alpha factor were included in the analysis in Figure 2G. Table S1 summarizes the microarray results. The raw data were submitted to GEO: GSE110704.

#### Quantitative proteomics data analysis

A compendium of in-house software was used to convert “.raw” files to mzXML format, as well as to correct monoisotopic m/z measurements and erroneous charge state assignments. Assignment of MS/MS spectra was performed using the Sequest algorithm. Database searching included all entries from the SGD (*Saccharomyces* Genome Database, March 20, 2015). Searches were performed using a 50 ppm precursor ion tolerance, and the product ion tolerance was set to 0.9Da. Trypsin protease specificity was required, allowing up to two missed cleavages. TMT tags on peptide N termini/lysine residues (+229.1629 Da) and carbamidomethylation of cysteine residues (+57.0215 Da) were set as static modifications while methionine oxidation (+15.9949 Da) was set as a variable modification. Peptide-spectrum matches (PSMs) were adjusted to a 1% false discovery rate (FDR). PSM filtering was performed as previously described (Huttlin et al., 2010) using an in-house linear discrimination analysis algorithm considering the following parameters: XCorr, peptide ion mass accuracy, charge state, peptide length and missed-cleavages. For TMT-based reporter ion quantitation, the signal-to-noise ratio (S:N) for each TMT channel was extracted and the closest matching centroid to the expected mass of the TMT reporter ion was identified. PSMs were identified, quantified, collapsed to a peptide FDR of 1%, and then collapsed further to a final protein level FDR of 1%. Protein assembly was guided by principles of parsimony to produce the smallest set of proteins necessary to account for all of the observed peptides using an in-house protein assembly algorithm. Peptide intensities were quantified by summing reporter ion counts across all matching PSMs using in-house software, as described previously (McAlister et al., 2012, McAlister et al., 2014). A 0.003 Th window around the theoretical *m/z* of each reporter ion was scanned, and the maximum intensity nearest the theoretical *m/z* was used. PSMs with MS3 spectra with TMT reporter summed signal-to-noise ratio < 100 were excluded from quantitation, and isolation specificity of > 0.7 was required (McAlister et al., 2012, Paulo et al., 2016). Protein quantitation values were exported for further analysis into Excel.

For further analysis only proteins with a minimal signal to noise ratio of 10 (average in 1 h time point) were considered. GSEA analysis was performed to compare the proteome of 3 h and 5 h arrested cells. A list of high quality GO terms (2023 terms, including cellular compartment, molecular function and biological processes) was downloaded from GO2MSIG and manually expanded to include a gene set of stress induced (ESR induced, 279 genes), stress repressed (ESR repressed, 586 genes) (Gasch et al., 2000) and transposable elements (91 genes). Genes were ranked based on signal to noise ratio and gene set permutation analysis was performed to determine false discovery rate.

### Data and Software Availability

#### Submitted data

Excel spread sheets of summarized Microarray, RNASeq and TMT-mass spectrometry data are available online with this article (Table S1. Microarray Analysis of Pheromone Response, Related to Figure 2, Table S2. S7 RNA Sequencing Data, Related to Figures 4, Table S3. Quantitative Proteomics Data, Related to Figure 4). The accession number for the microarray data and RNA Seq data reported in this paper is GEO: GSE110704. Raw proteomic data are available upon request.

### Additional Resources

GO annotation file for GSEA analysis was downloaded from: http://www.go2msig.org/cgi-bin/prebuilt.cgi?taxid=559292GO

## Supplemental Information

Table S1. Microarray Analysis of Pheromone Response, Related to Figure 2

Table S2. S7 RNA Sequencing Data, Related to Figures 4

Table S3. Quantitative Proteomics Data, Related to Figure 4

Table S4. Related to STAR Methods
